# Automated tracking of tumor-stroma morphology in microtissues identifies functional targets within the tumor microenvironment for therapeutic intervention

**DOI:** 10.18632/oncotarget.5046

**Published:** 2015-09-03

**Authors:** Malin Åkerfelt, Neslihan Bayramoglu, Sean Robinson, Mervi Toriseva, Hannu-Pekka Schukov, Ville Härmä, Johannes Virtanen, Raija Sormunen, Mika Kaakinen, Juho Kannala, Lauri Eklund, Janne Heikkilä, Matthias Nees

**Affiliations:** ^1^ Turku Centre for Biotechnology, University of Turku, Turku, FI-20520, Finland; ^2^ VTT Technical Research Centre of Finland, Turku, FI-20521, Finland; ^3^ Centre for Machine Vision Research, University of Oulu, Oulu, FI-90014, Finland; ^4^ Department of Mathematics and Statistics, University of Turku, Turku, FI-20014, Finland; ^5^ University Grenoble Alpes, iRTSV-BGE, Grenoble, F-38000, France; ^6^ CEA, iRTSV-BGE, Grenoble, F-38000, France; ^7^ INSERM, BGE, Grenoble, F-38000, France; ^8^ Institute of Biomedicine, University of Turku, Turku, FI-20520, Finland; ^9^ Biocenter Oulu and Department of Pathology, University of Oulu and Oulu University Hospital, Oulu, FI-90220, Finland; ^10^ Oulu Center for Cell-Matrix Research, Biocenter Oulu and Faculty of Biochemistry and Molecular Medicine, University of Oulu, Oulu, FI-90014, Finland

**Keywords:** 3D co-culture, cancer associated fibroblast (CAF), phenotypic screening, invasion, focal adhesion kinase (FAK)

## Abstract

Cancer-associated fibroblasts (CAFs) constitute an important part of the tumor microenvironment and promote invasion via paracrine functions and physical impact on the tumor. Although the importance of including CAFs into three-dimensional (3D) cell cultures has been acknowledged, computational support for quantitative live-cell measurements of complex cell cultures has been lacking. Here, we have developed a novel automated pipeline to model tumor-stroma interplay, track motility and quantify morphological changes of 3D co-cultures, in real-time live-cell settings. The platform consists of microtissues from prostate cancer cells, combined with CAFs in extracellular matrix that allows biochemical perturbation. Tracking of fibroblast dynamics revealed that CAFs guided the way for tumor cells to invade and increased the growth and invasiveness of tumor organoids. We utilized the platform to determine the efficacy of inhibitors in prostate cancer and the associated tumor microenvironment as a functional unit. Interestingly, certain inhibitors selectively disrupted tumor-CAF interactions, e.g. focal adhesion kinase (FAK) inhibitors specifically blocked tumor growth and invasion concurrently with fibroblast spreading and motility. This complex phenotype was not detected in other standard *in vitro* models. These results highlight the advantage of our approach, which recapitulates tumor histology and can significantly improve cancer target validation *in vitro*.

## INTRODUCTION

The major difficulty when developing cancer therapeutics is the limited availability of biologically relevant and predictive *in vitro* models for chemosensitivity tests, target validation and high content phenotypic screening. The challenge is to develop cell culture models that better resemble cancer tissues, and more faithfully recapitulate the complex architecture of tumors *in vivo*. The vast majority of cell-based assays used for testing cancer drug efficacy are based on growing cells as two-dimensional (2D) monolayers on plastic, or as three-dimensional (3D) floating spheroids in suspension [1]. The biology and homeostasis of tumors, however, is strongly influenced by the tumor microenvironment. This requires novel, cell- and tissue-based assays that reflect the effects of extracellular matrix (ECM), cell-cell contacts, cell-matrix interactions, and recapitulate the tissue architecture of cancers. Embedded 3D cell culture techniques strictly avoid plastic surfaces, thus allowing cells to modulate the ECM [2–4]. Drug sensitivity of cancer cells observed in 2D monolayer cultures can be very different from 3D cell cultures. It is widely accepted that 3D organoids reflect *in vivo* growth of epithelial tumor cells more reliably and provide better readouts for drug testing [2, 5, 6]. The broad spectrum of phenotypic changes observed upon drug exposure can be utilized as a sensitive readout for measuring compound efficacy.

In the tumor microenvironment, a variety of stromal cell types are present. Cancer-associated fibroblasts (CAFs) are the most abundant stromal cell type in carcinomas, and play a prominent role in tumor growth and progression. CAFs secrete a plethora of growth factors, cytokines and chemokines, which stimulate growth, invasive and metastatic processes. CAFs participate in the cross-talk with tumor cells, are recruited by cancer cell-secreted factors like TGFβ and PDGF, and lead the way for tumor cell invasion [7, 8]. In addition, CAFs have a strong physical impact on the tumor tissue, resulting in ECM remodeling, contraction and increased tumor stiffness [9, 10]. Rather than operating as single cellular units, CAFs merge to form stromal collective cohorts or syncytia. In order for fibroblasts to propagate syncytial behavior, a coordinated cell adhesion program is conducted [11, 12], which shapes cancer tissue morphologies. This collective configuration allows CAFs to form a defined cancer cell niche and coordinate contractile and migratory behavior, and assists in the induction of epithelial-to-mesenchymal transition (EMT) at the tumor edges [13, 14]. It is currently only poorly understood if and how stromal and tumor cells form direct cell-cell-interactions, and how these may contribute to the tumor biology.

Although the significance of adding stromal cells to 3D cell cultures to model heterotypic cell–cell interactions has long been acknowledged, the practical implementation of standardized co-cultures that include multiple cell types remains demanding. Optimal culture conditions that allow each cell type to grow and maintain in stable homeostasis with each other are difficult to establish. The major challenge regarding complex 3D cell cultures is the detailed analysis of the experiments, including segmentation and tracking of cell movements as well as the analysis of their distinct morphologies [3, 15]. Most analyses of 3D cultures that include stromal components only provide poorly informative growth curves from generalized fluorescent measurements or impedance, sometimes combined with incidental, molecular snapshots by immunofluorescence (IF) end-point staining [16–21]. Alterations in stromal motility and tumor cell plasticity are difficult to measure and usually ignored. To obtain quantitative cell tracking of dynamic biological processes involved in tissue formation, invasion, growth and drug response, novel computational methods are needed that provide real-time automatic measurements of complex cellular interactions and phenotypic changes. Several studies have utilized automatic analysis of time-lapse videos [22], and both commercial and open software tools are available for automated live-cell analysis of monocultures [23–25]. However, computational support for quantitative live-cell tracking and morphological measurements of complex tumor microtissues embedded in ECM is currently lacking.

In this study, we established stable and reproducible microtissues of prostate cancer (PrCa) cell lines in combination with CAFs, embedded in biologically relevant ECM. Our novel computational analysis pipeline was simultaneously used for quantification of morphological changes, and monitoring of cell motility in 3D cancer co-culture models in real-time. These microtissues enable evaluation of treatments with perturbants, using live-cell imaging and tracking of fibroblast and tumor organoid dynamics over several weeks in an automated fashion. A panel of small molecule inhibitors was utilized to challenge the model system and affect the nature of direct and indirect (paracrine) tumor-CAF interactions. In particular, focal adhesion kinase (FAK) inhibitors simultaneously affected both tumor and stromal compartments in 3D co-culture, which was neither detectable in 3D monoculture, nor in 2D settings. FAK inhibitors specifically reduced tumor growth and invasiveness. This analysis approach allows continuous monitoring and quantification of CAF-promoted tumor cell growth and invasion, and facilitates more comprehensive *in vitro* cancer target validation strategies that integrate differentiation, plasticity and homeostasis of cancer tissues.

## RESULTS

### Automated segmentation, quantification and tracking of 3D co-culture morphologies

**Figure 1:**
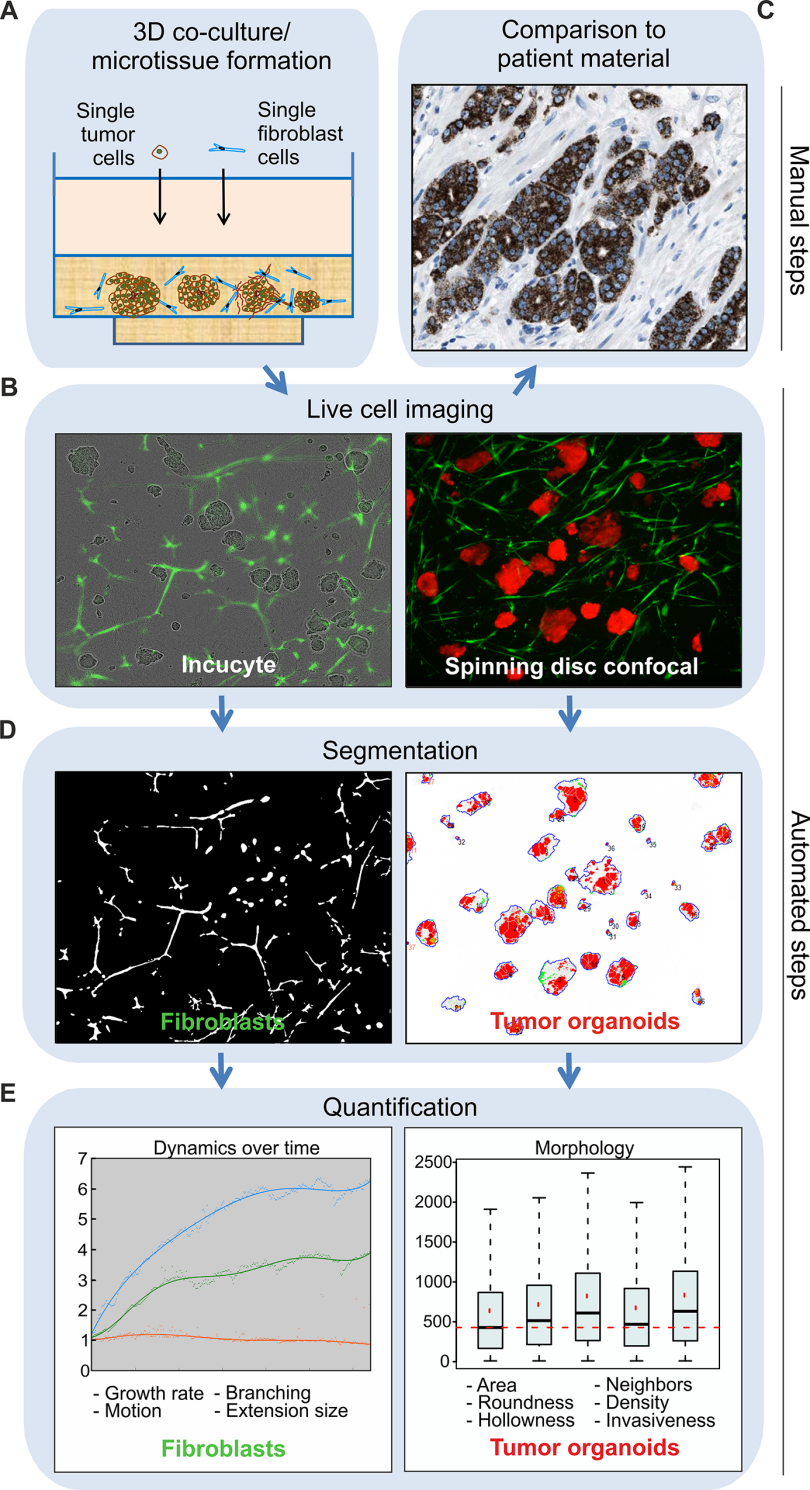
Tracking of tumor-stroma morphology and dynamics in microtissues by automated image analysis Schematic presentation of the computational analysis pipeline on example image data. **A.** Manual establishment of tumor-fibroblast co-cultures generated from traceable cell lines in the standardized and miniaturized experimental platform, allowing cells to be embedded in a narrow focal plane of ECM. **B.** Real-time live-cell imaging is employed by continuous monitoring for up to several weeks. Incucyte FLR phase contrast and green fluorescent images are captured, ideal for investigations of long dose-responses and time-course experiments of fibroblast dynamics in the co-cultures (left panel). Spinning disc confocal microscopic fluorescent live-cell images of tumor cells together with fibroblasts are used for detailed examination of tumor organoid morphology (right panel). **C.** The tissue architecture of the resulting microtissues can be empirically compared to clinical samples, in order to assess how consistently *in vivo* tumor morphology observed in patients is recapitulated. This reference image displays alpha-methylacyl-CoA racemase (AMACR) antibody staining of PrCa tissue (www.proteinatlas.org), which is a widely used biomarker in PrCa. **D.** Incucyte FLR images sequences are used for fibroblast segmentation, achieved by local adaptive thresholding (left panel). Confocal microscopy images are used for segmentation of tumor organoids carried out by AMIDA (right panel). **E.** Final quantification is based on dual segmentation of tumor and fibroblast cells. Growth, motion and shape analyzes of fibroblasts are performed over time. In addition, detailed morphological measurements of tumor organoids are performed at the end-point. Example graphs are displayed.

To model the heterotypic cell–cell interactions between tumor and stroma, and to explore how fibroblasts affect the tumor growth and morphology over time, an automated image analysis approach for tracking 3D co-cultures in real-time was established (Figure 1). We generated miniaturized tumor-fibroblast co-cultures in ECM that can be perturbed and observed by live-cell microscopy (Figure 1A–1B), in order to mimic complex tumor architecture (Figure 1C). The microscopic images were segmented, using different adaptations for both cell types. For fibroblast sequences, local adaptive thresholding was used [26], while tumor organoids were traced using our automated image data analysis software (AMIDA) [27] (Figure 1D). Fibroblasts were quantified from fluorescent phase contrast image sequences to track dynamic changes for over two weeks. Quantification of tumor organoids from maximum projection confocal images was performed to detect phenotypic changes in response to molecular perturbations (Figure 1E). This analysis pipeline allows simultaneous automated imaging, segmentation and quantification of both cell compartments (Figure 1). It can be utilized for functional cancer target validation, chemosensitivity testing or high-content screening purposes, when the impact of tumor/stroma interactions is investigated.

### Establishment of complex 3D co-culture cancer models that allows imaging

Real-time tracking of complex co-culture models requires distinction of the two counterparts. Therefore, traceable cell lines that stably expressed fluorescent markers (GFP; fibroblast and dsRed; tumor cells) were applied. For the 3D co-cultures, we utilized a standardized and miniaturized protocol [2, 27]. All 3D experiments were performed in 96-well angiogenesis plates (Ibidi), exhibiting a well-in-a-well geometry that consists of two compartments. The method allows cells to be embedded in a narrow focal plane embedded between two layers of ECM. Single cell suspensions were mixed with ECM at a density of 700–1500 cells/well, depending on the cell line. Hence, one tumor organoid is typically generated from a single cell (Supplementary Figure S1A), and several hundreds of organoids are formed per well. To generate stable, reproducible and complex co-cultures, serum (FBS) concentrations and cell ratios were optimized and standardized (Supplementary Figure S1B, see Materials and Methods). Next, untransformed prostate (EP156T) and transformed PrCa cell lines (LNCaP, VCaP, PC3), were co-cultured with PF179T CAFs [28], using the optimized settings (2% FBS, tumor:stroma ratio 2:1). All cells were cultured in three types of ECM: Matrigel, collagen type-I, or a 1:1 mixture of both. The PF179T CAFs formed elongated fibroblast-like structures in ECM-containing collagen, but remained small and round in Matrigel (Supplementary Figure S1C). Of the tumor cells, LNCaP cells generally formed rounded organoids in all ECM preparations and 3D settings. In contrast, single VCaP cells did not proliferate under any 3D conditions, not even in the presence of fibroblasts. To facilitate the growth of VCaP in embedded 3D conditions, cells were pre-cultured as floating spheroids in low-attachment culture, prior to transfer into ECM (see Materials and Methods). After 3–5 days, floating spheroids were transferred into the respective ECM and gave rise to proliferating organoids (Supplementary Figure S1C). Untransformed EP156T cells and transformed PC3 cells both generated branching and invasive phenotypes respectively, which appeared macroscopically very similar (Supplementary Figure S1C). The addition of CAFs to PC3 cells resulted in loss of organoid structure, and thus prohibited the use of these cells for studies on the invasive properties of cancer cells in 3D co-cultures. In order to recapitulate the characteristic *in vivo* morphology of tumor islands surrounded by stroma, we focused the tissue-model development on LNCaP cells.

### Tracking and quantification of fibroblast dynamics reveals the importance of collagen for CAF cohort spreading

**Figure 2:**
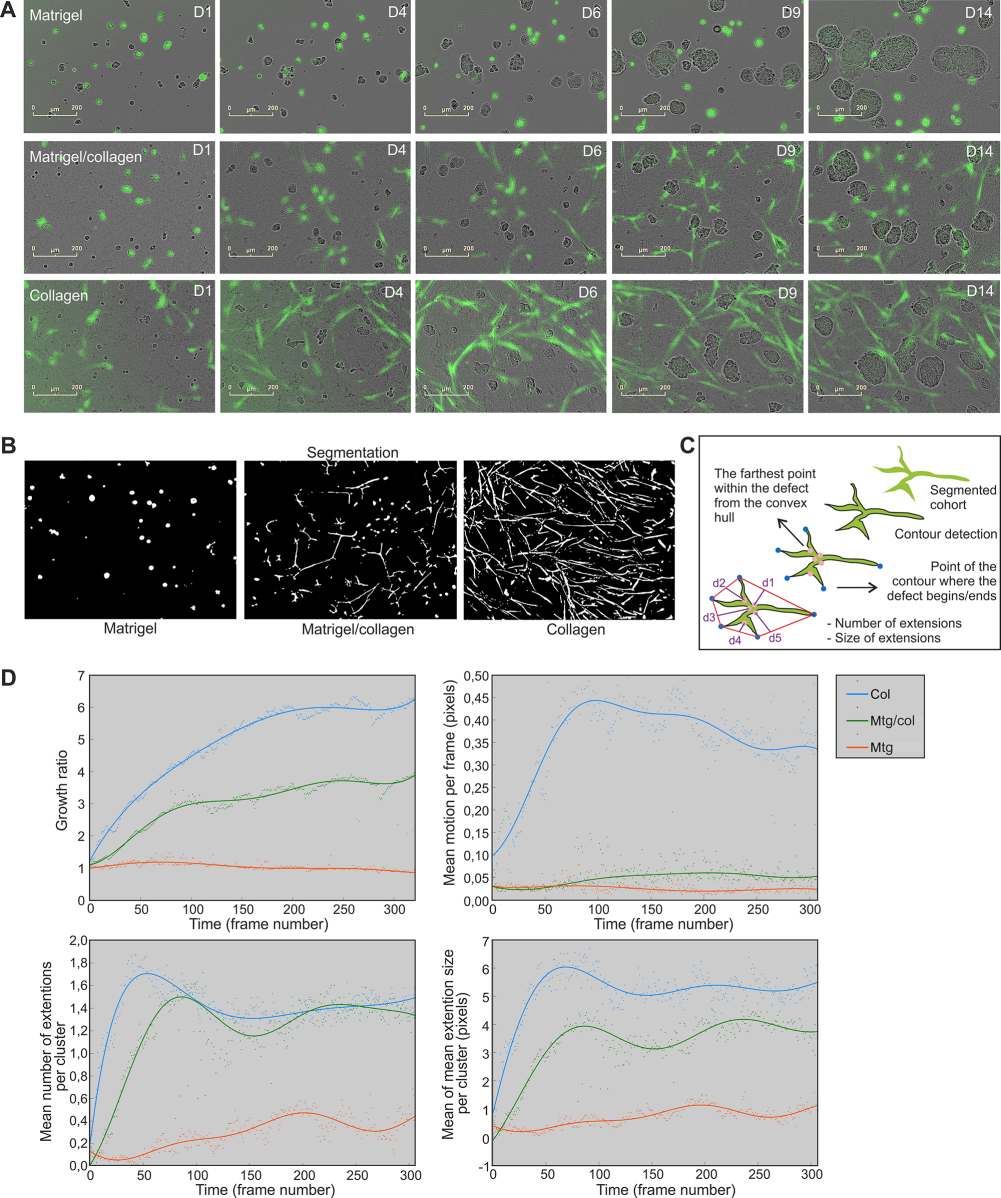
Collagen is required for CAF cohort growth, movement and branching in 3D co-cultures **A.** Incucyte FLR real-time live-cell imaging of LNCaP organoids (phase contrast) and PF179T CAFs (green) co-cultured for 14 days in Matrigel, Matrigel/collagen mixture and collagen. Scale bar: 200 μm. **B.** Fibroblast morphology and dynamics was analyzed from the time-lapse images in an automated fashion. CAF cohort segmentation was performed by local adaptive thresholding and example images of segmented CAFs in three matrices are described. **C.** Schematic drawing depicts how branching of each CAF cohort was assessed by extracting the closed contour and detecting the convex hull of the contour as well as contour convexity defects. **D.** Quantification of fibroblast growth rate (area), motion per frame, number of extensions per cluster and extension size per cluster, is shown over time. Every dot represents a value per each time frame of the image sequence. Segmented real-time live-cell image sequences from three different ECMs were used for quantification. Col: collagen, Mtg/col: Matrigel/collagen 1:1 mixture, Mtg: Matrigel.

Next, we investigated how tumor cells and fibroblasts assemble in different types of ECM and generate functional microtissues over time. Real-time live-cell imaging was carried out in a systematic fashion. The Incucyte FLR fluorescent live-cell imaging system was used for continuous tracking of long-term dose-response and time-course experiments in 3D co-cultures. Interestingly, when culturing LNCaP tumor cells together with PF179T stromal cells in different ECM preparations, drastic differences in fibroblast dynamics were observed (Figure 2). In Matrigel, CAFs remained small and rounded, but nevertheless actively moved towards the tumor organoids (Figure 2A upper panel, Supplementary Video S1A). When co-cultured in collagen, CAFs became elongated and very motile, and actively generated a multicellular stromal network surrounding the tumor organoids (Figure 2A lower panel, Supplementary Video S1B). In Matrigel/collagen mixture, CAFs aligned into multi-cellular syncytia (Figure 2A middle panel, Supplementary Video S1C). In the presence of pure collagen, CAF cohorts were able to remodel the ECM and utilize collagen fibers for traveling, thus illustrating that the presence of collagen fibers is essential for fibroblast dynamics and morphology. Notably, CAFs grown in the absence of tumor cells did neither spread, nor form syncytia and survived only poorly in 3D culture (Supplementary Figure S1D). These results emphasize the complex nature of the tumor-stroma interdependency.

For quantification of fibroblast dynamics, Incucyte FLR time-lapse image sequences were analyzed in an automated fashion (Figure 2B, Supplementary Figure S2A). Image sequences were first stabilized using frame-by frame alignment (Supplementary Video S2), and filtered using median filtering and non-local means de-noising (Supplementary Figure S2B). Sequences were then segmented using local adaptive thresholding (Figure 2B, Supplementary Video 3) [26]. Growth, motion and shape analyzes were then performed based on the initial segmentation (Figure 2B). The growth of CAF cohorts was associated with the size of the segmented region i.e. total number of pixels in the fluorescent image covered by fibroblasts, as a function of time (Figure 2B). Motion analysis of CAF cohorts was implemented by using dense optical flow estimation [29] (Supplementary Figure S2C, Supplementary Video S4). The shape analysis, i.e. branching, of each CAF cohort was examined by extracting the closed contour, and detecting the convex hull of the contour together with contour convexity defects (Figure 2C, Supplementary Figure S2D, Supplementary Video S5). The number of extensions and mean extension size per cluster was used to illustrate branching, separately for each cluster. The means of these values were then plotted over time. Lastly, LNCaP organoids were also segmented (Supplementary Figure S2E) and growth (area) was quantified from the same sequences (Supplementary Figure S2F). All algorithms are described in the Materials and Methods section.

We found that PF179T cells grew fastest in pure collagen, not at all in Matrigel, and at an intermediate rate in the Matrigel/collagen mixture (Figure 2D). Matrigel influenced fibroblast motility in a dominant negative fashion, as CAFs were very motile only in pure collagen matrix (Figure 2D). The mean number of extensions of fibroblast cohorts was affected in a dominant positive fashion by collagen, as PF179Ts generated extensions in both collagen and mixture (Figure 2D) but not in pure Matrigel. The extension size per cohort showed that CAFs in Matrigel/collagen mixture displayed an intermediate phenotype, positioned between collagen and Matrigel (Figure 2D), similar to LNCaP tumor growth (Supplementary Figure S2F). Taking together, our approach to track and quantitate the morphology of fibroblast syncytia surrounding tumor organoids revealed that collagen fibers are essential for CAF cohorts to grow, move and spread. This method allows automated analysis of a large number of low-resolution time-lapse image sequences to be quantitated, potentially as a measure for tumor stiffness [30].

### CAF-promoted invasive properties of tumor organoids

**Figure 3:**
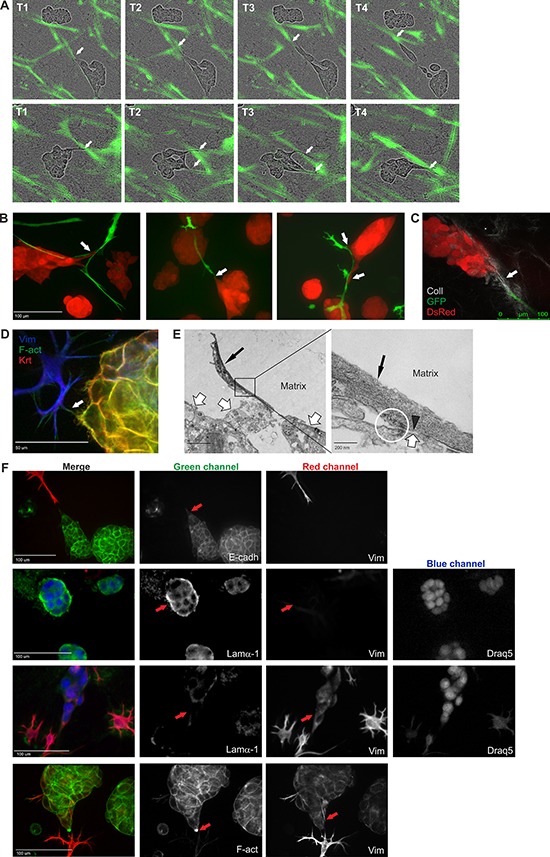
CAFs remodel the ECM and facilitate tumor cell invasion **A.** Two example sequences of Incucyte FLR real-time live-cell imaging of LNCaP cells (phase contrast) and PF179T CAFs (green), co-cultured in Matrigel/collagen mixture. CAFs appear to pull the matrix and lead the way for tumor cells to invade. White arrows depict tumor-stroma interaction. T: timepoints, captured with 4 h intervals. **B.** Confocal live-cell images of tumor stroma interactions. Tumor cells follow the fibroblast tracks. Red: dsRed-LNCaP, green: GFP-PF179T CAFs. White arrows depict tumor-stroma interaction. Scale bar: 100 μm. **C.** Second-harmonic generation microscopy image of collagen-mediated tumor-CAF connection. Red: dsRed-LNCaP, green: GFP-PF179T CAFs, grey: collagen fibers. Scale bar: 100 μm. **D.** IF staining implying direct contact between tumor and stromal cells. Arrow depicts tumor-stroma interaction. Vimentin (Vim): mesenchymal cell marker, Pan-keratin (Krt): epithelial cell marker, F-actin (F-Act). Scale bar: 50 μm. **E.** Left panel: high-resolution transmission electron microscopy (TEM) images, showing direct tumor and stromal cell contact. Thin black arrow depicts α-smooth muscle actin (α-SMA)-rich CAF, thick white arrow depicts tumor organoid. Scale bar: 2 μm. Right panel: Marked area in the left panel is blown up. Black arrowhead points to α-SMA-rich cytoskeleton in the CAF, directly adjacent to the tumor cell membrane. The circle highlights electron dense material, indicating cortical actin accumulation, a hallmark of cell-cell contacts. Scale bar: 200 nm. For the complete TEM panel, see Supplementary Figure S3. **F.** Representative IF-stainings displaying CAF-promoted tumor invasion. Tumor cells lose both the E-cadherin expression (panel 1) and the basal lamina (panel 2 and 3) in the organoid protrusion, in response to nearby CAFs starting to pull the matrix. Instead, the tumor protrusion expressed vimentin (panel 3 and 4) and an accumulation of actin is observed in the invasive tip (panel 4). Vimentin (Vim): mesenchymal cell marker, E-cadherin (E-cadh): epithelial cell marker, Laminin-α1 (Lamα1): basal lamina, F-actin (F-Act), Draq5: nuclear stain. Red arrows depict the important details. The separate channels are displayed in black and white. All channels are shown in color in the merged images. Scale bar: 100 μm.

In order to establish in more detail how CAFs affect and communicate with the tumor organoids in the 3D co-cultures, we performed high-resolution microscopy. In collagen-containing matrix, the CAFs appeared to pull the organoids while generating motion tracks in the matrix for single tumor cells to follow or occasionally even break free from the organoid structures (Figure 3A–3B, Supplementary Video S1C). Second-harmonic generation live-cell microscopy revealed that stromal syncytia were able to attach to single tumor cells, often with the help of collagen fibers, followed by movement of the organoid body into the same direction (Figure 3C). Collagen reorganization at the tumor-stromal interface has been described *in vivo* and shown to facilitate local invasion [31, 32]. Interestingly, direct cell-cell contacts between tumor and stromal cells was also observed (Figure 3D–3E). Electron-dense material and cortical actin accumulation was detected at spots where α-smooth muscle actin (α-SMA)-rich CAFs were touching neighboring tumor cell membrane (Figure 3E, Supplementary Figure S3). This is a hallmark of cell-cell contacts [33, 34]. To investigate if the CAF-mediated protrusions of the tumor organoids actually are invasive, immunofluorescence (IF) stainings were conducted. When no CAFs were closely situated to the LNCaP organoids, they did not express mesenchymal markers like vimentin, but formed solid round structures surrounded by an intact basal lamina, which prohibited tumor cells to escape from the organoid (Figure 3F). In stark contrast, when CAFs were in close proximity of the tumor organoid and pulled the matrix away from the tumor organoid, the basal lamina almost completely disintegrated. At these sites of intense interaction, the tumor cells in the protrusion had undergone EMT, and were expressing vimentin (Figure 3F). Furthermore, a marked enrichment of actin at the tips of organoid protrusions was discovered (Figure 3F), which is an established indication of invadopodia [35]. Taken together, our results revealed that fibroblasts promote formation of invadopodia, and that the complex co-culture model recapitulate CAFs-led invasion, a previously described phenomenon [7, 8].

**Figure 4:**
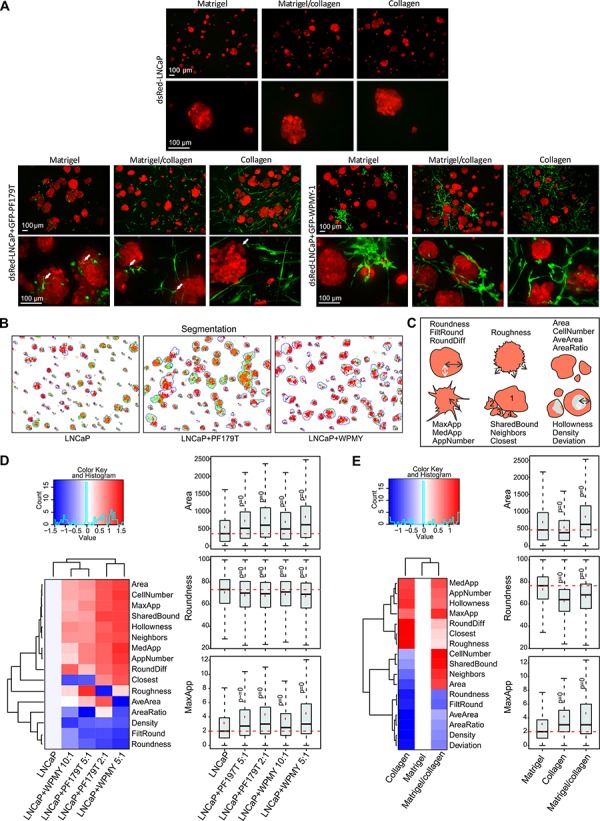
Addition of fibroblasts promote growth and invasiveness of tumor organoids **A.** Representative maximum projection confocal images of 3D mono and co-cultures cultured for 14 days in Matrigel, collagen and mixture. Red: dsRed-LNCaP, green: GFP-PF179T (left panel), GFP-WPMY-1 (right panel). White arrows in left, lower panel depict filopodia-like structures of PF179T CAFs interacting with tumor organoids. Scale bar: 100 μm. **B.** LNCaP organoid segmentation was performed by the automated image data analysis software AMIDA [27], and example images of segmented LNCaP organoids in in Matrigel/collagen 3D mono- or co-culture are described. Red: dsRed LNCaP segmentation, green: overlapping GFP signal. **C.** Schematic drawing depicts morphological parameters that were analyzed with AMIDA. **D.** Classification of morphological changes in tumor organoids by addition of different fibroblast ratios (PF179T and WPMY-1), in comparison to LNCaP monoculture. **E.** Classification of morphological changes in tumor organoids co-cultures in different ECM; collagen and Matrigel/collagen mixture in comparison to Matrigel. (D–E) Heatmaps summarize altered parameters from AMIDA analysis. Values are color-coded as red (increased) versus blue (decreased), relative to the control. For explanation of morphometric parameters, see Supplementary Table S1. Box and whisker plots shown for selected parameters from the heatmap. Area describes the size of the segmented structure in pixels, Roundness is a measure of loss of the round organoid phenotype, and MaxApp is an estimate for the maximum length of appendages (invasive processes) observed in the segmented structure. *P*-values are Bonferroni-corrected from Mann-Whitney *U*-test and compared to control. *P* = 0 indicates *P*-values < 0.001.

Next, tumor-stroma interactions in different ECMs were investigated. Higher resolution, confocal images of living dsRed-LNCaP and GFP-PF179T 3D co-cultures were acquired at day 14. The matrix affected shape, size and texture of tumor organoids in co-culture (Figure 4A, lower panels). Although the morphology and spreading of PF179T cells diverged drastically in distinct ECMs, fibroblasts were able to form small filopodia-like structures in all matrices (Figure 4A, left panel, Supplementary Video S1A–1C). For comparison, another fibroblast cell line, GFP-WPMY-1 [36], was used in co-culture with dsRed-LNCaP. Due to rapid proliferation of transformed WPMY-1 cells, only a minute amount of stromal cells was necessary (tumor:stroma ratio 10:1) to result in a stable microtissue architecture (Figure 4A, right panel, Supplementary Figure S4). IF-stainings revealed evident morphological differences between these two CAF lines; PF179T cells formed many direct interactions with the tumor organoids via filopodia (Supplementary Figure S5, Supplementary Video S6), whereas WPMY-1 cells mainly surrounded the organoids and formed no or few cell contacts. No significant apoptosis was detected in either type of microtissue, only single tumor cells were occasionally undergoing apoptosis (Supplementary Figure S6). In addition, pre-formed RFP-VCaP spheroids were co-cultured with GFP-PF179T fibroblasts in different matrices (Supplementary Figure S7). Both LNCaP and VCaP 3D co-cultures generated standardized, stable, and reproducible microtissues, ideal for drug target validation and phenotypic assays.

To assess how the addition of fibroblasts affects the growth and morphology of tumor organoids, we utilized AMIDA, developed for high-content phenotypic analysis of 3D cultures [27]. AMIDA enables rapid segmentation and quantitative measurements of a large number of images, typically containing hundreds of organoids with a spectrum of different shapes, sizes and textures (Figure 4B–4C). This computational read-out represents a compromise between detailed imaging of morphological details, and fast experimental throughput. Significant, but subtle differences in key morphometric parameters were discovered when fibroblasts were added to LNCaP organoids (Figure 4D, Supplementary Figure S8, Supplementary Table S1). Addition of both fibroblast lines (PF179T and WPMY-1) stimulated the growth of tumor organoids (Area, CellNumber), likely due to secreted growth factors. The presence of CAFs also induced a loss of cell-cell-contacts and reduced epithelial differentiation, typically measured by the less rounded shape of organoids (Roundness, FiltRound), or reduced structure density (Density), a previously described characteristic of epithelial or acinar differentiation [2, 37]. Concomitantly, a significant increase in the number (AppNumber) and length (MaxApp, MedApp) of “appendages” or invasive protrusions originating from the multicellular organoids was observed (Figure 4D), established as early signs of tumor cell invasion in 3D settings (Figure 3). Alterations in morphometric parameters of LNCaP tumor organoids were also discovered when co-cultured with CAFs in different matrices (Figure 4E, Supplementary Figure S8). Organoids grew largest in the Matrigel/collagen mixture (Area, CellNumber). Addition of collagen affected pre-invasive properties of organoids, resulting in increased number (AppNumber) and length (MaxApp, MedApp) of the invadopodia (Figure 4E). In summary, a mixture of Matrigel (laminin) and collagen resulted in optimal growth conditions, supporting growth of both tumor and stromal cells. Our image analysis approach demonstrates the possibility to visualize and quantify organoid changes associated with fibroblast-promoted tumor invasion.

### FAK inhibitors constrain both tumor growth and spreading of stromal cells

To investigate the potential of our platform for functional target validation and provide biologically significant, phenotypic readout, we performed chemical perturbation of selected proteins that are associated with tumorigenesis; wingless-type MMTV integration site family (WNT), β-catenin signaling, and γ-secretase protein complex (Notch) [38], FAK [39], AKT kinase (AKT), phosphoinositide-3-Kinase (PI3K) and mammalian target of rapamycin (mTOR) [40]. We also selected granulocyte-macrophage colony-stimulating factor (GM-CSF), which has previously been described as active in tumor progression [41] (Table 1). We were specifically interested to explore how perturbation of these molecules may affect tumor and stroma as a single functional unit, and thereby block tumor growth as a whole. To investigate the morphometric effect of selected compounds on PrCa, dsRed-LNCaP cells were co-cultured with PF179T CAFs in Matrigel/collagen mixture for 14 days. Compounds were added to the medium after 4 days, during which multicellular tissue structures had already formed.

**Table 1:** Compounds used for perturbation of 3D co-cultures

Abbreviation	Function
DMSO	Control
GSI-953	Begacestat, γ-secretase inhibitor
IWP-2	Inhibitor of WNT processing and secretion
FH-535	Inhibitor of WNT/β-catenin signaling
GM-CSF	Granulocyte-macrophage colony-stimulating factor
Y11	Inhibitor of focal adhesion kinase (FAK)
PF-573228	Inhibitor of FAK
10-DEBC	Inhibitor of AKT/PKB
PI-103	Inhibitor of DNA-PK, PI3-kinase (p110α) and mTOR
LY-294002	Inhibitor of PI3-kinase

**Figure 5:**
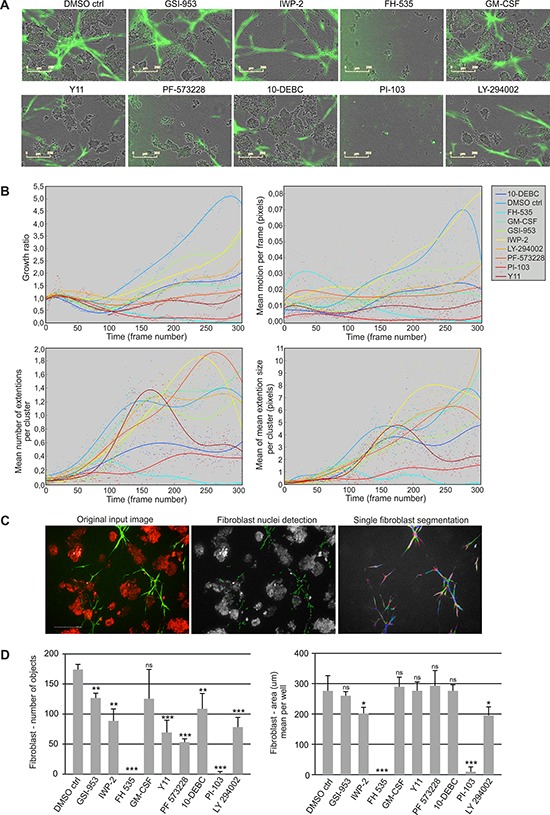
Small molecule inhibitors block growth and dynamics of fibroblasts in 3D co-culture **A.** Representative live-cell images of LNCaP (phase contrast) and GFP-PF179T cells (green) co-cultured in Matrigel/collagen mixture, treated with inhibitors (3 μM) or GM-CSF (0.3 ng/ml). For description of perturbants, see Table 1. Scale bar 200 μm. **B.** Quantification of perturbants’ effects on fibroblast growth rate (area), motion per frame (motility), extension size per cluster and number of extensions per cluster (branching), is shown over time. Segmented real-time live-cell image sequences were used for the quantification. Every dot represents a value per each time frame of the image sequence. **C.** Segmentation of single fibroblasts using dual labeled, Histone 2B-mCherry/GFP-PF179Ts, together with dsRed-LNCaP cells. All the fibroblast nuclei (green: mCherry) were selected and their surrounding cytoplasm was detected by the overlay of the GFP signal from the fibroblasts (shown in different colors). The segmentation was performed using the PE Harmony software. **D.** Quantification of the effect of perturbants on single fibroblast number and area. Spinning disc confocal images taken at the end point (day 14) were used for quantification. Error bars depict SD. *P*-values * *P* < 0.05, **P < 0.01, *** *P* < 0.001, ns: not significant, by Student *t*-test.

The growth and morphology of fibroblasts and tumor organoids was investigated in 3D co-cultures using live-cell imaging and tracking (Figure 5A). Inhibition of WNT/β-catenin signaling (FH-535) and PI3K/mTOR (PI-103) displayed cytotoxic effects on the CAFs already at day 8, thus effectively killing fibroblasts completely by day 11 (Supplementary Figure S9A). Interestingly, FAK inhibition (Y11, PF-573228) most prominently reduced fibroblast growth compared to control treatment, without strong cytotoxic effects and without elimination of all the CAFs, (Figure 5B, Supplementary Figure S9A, Videos S7A–E). The inhibitor LY294002, although also targeting PI3K, only showed intermediate effects. The same molecules were also important for fibroblast movement, as corresponding inhibitors repressed CAF motility over time. Examination of the extension size and number of the CAF cohorts revealed that inhibition of WNT/β-catenin, PI3K, AKT and FAK caused substantial alterations (Figure 5B). Inhibitors FH-535 (WNT/β-catenin) and PI-103 (PI3K/mTOR) effectively removed fibroblast extensions due to killing of CAFs, whereas 10-DEBC (AKT) and Y11 (FAK) only decreased CAF cohort branching (Figure 5B). For single fibroblast analysis, we imaged dual labeled GFP/Histone B2-mCherry-PF179T cells together with dsRed-LNCaP cells using the PE Operetta High Content Imaging System, combined with PE Harmony High Content Imaging and Analysis Software (Figure 5C). Histone B2-labeled CAFs enabled single cell segmentation and showed that the number of fibroblasts was only reduced with Y11, PF-573228 (FAK) and LY-294002 (PI3K) inhibitors, while FH-535 (WNT/β-catenin) and PI-103 (PI3K/mTOR) both effectively abolished all the CAFs (Figure 5D). More importantly, the size (Area) of the living, single fibroblasts was not significantly affected by FAK perturbation, whereas it was drastically decreased by use of the cytotoxic WNT and PI3K/mTOR inhibitors, and thereby indicating death of CAFs (Figure 5D).

**Figure 6:**
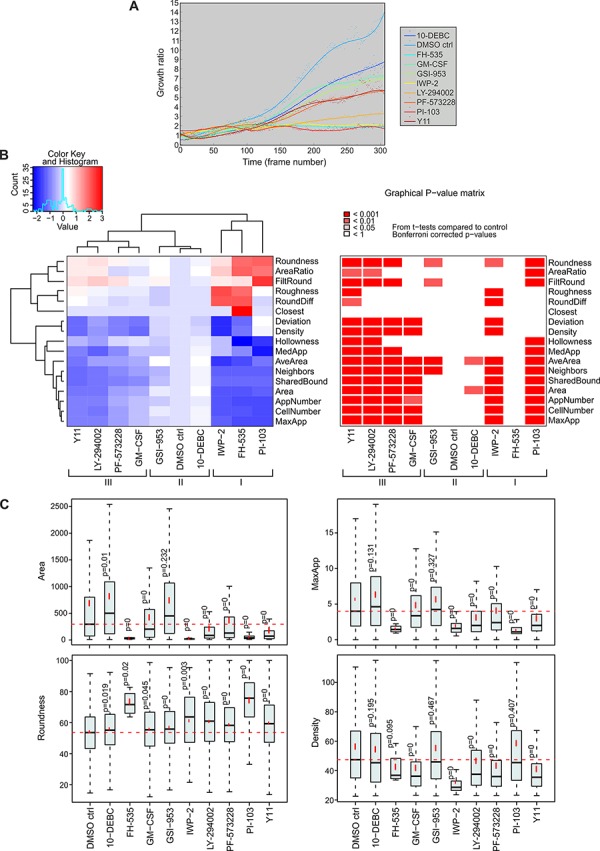
Inhibition of cancer-associated molecules affects growth and morphology of tumor organoids in 3D co-culture **A.** LNCaP tumor organoid growth was analyzed from Incucyte FLR time-lapse images in an automated fashion. Quantification of tumor fibroblast growth rate (area) is shown over 14 days. **B.** Classification of morphological changes in tumor organoids by the use of small molecule inhibitors (3 μM) or GM-CSF (0.3 ng/ml). Three distinct groups of tumor phenotypes are marked with roman numerals (I, II and III). Heatmap and graphical *P*-value matrix summarizes the altered parameters from the AMIDA analysis. Values are color-coded as red (increased) and blue (decreased), relative to the control. For explanation of morphometric parameters, see Supplementary Table S1. *P*-values are Bonferroni-corrected from *t*-tests and compared to DMSO control treatment (DMSO ctrl). **C.** Box and whisker plots of selected parameters from the heatmap. Area describes the size of the segmented structure in pixels, Roundness is a measure of loss of the round organoid phenotype, in percentage, MaxApp is an estimate for the maximum length of appendages of the segmented structure, and Density is measured as the intensity of the red channel for the segmented structure. *P* = 0 indicates *P*-values < 0.001.

In parallel to the CAF analyses, tumor organoid growth in 3D co-cultures was investigated using live-cell tracking. Reduced organoid growth was detected with the following inhibitors: PI-103 (PI3K/mTOR), LY-294002 (PI3K), IWP-2 (WNT), FH-535 (WNT/β-catenin), Y11 and PF-573228 (FAK) (Figure 6A). We continued with end-point morphometric analysis of tumor organoids co-cultured with CAFs (Supplementary Figure S9B). Blocking of molecules associated with carcinogenesis resulted in significant changes of tumor morphology, and compounds were classified into three groups based on the tumor phenotype (Figure 6B). Inhibitors FH-535 (WNT/β-catenin), IWP-2 (WNT) and PI-103 (PI3K/mTOR) effectively eliminated tumor cells/organoids. IWP-2 specifically abolished the tumor cells without affecting the stromal cells. In contrast, GSI-953 (γ-secretase) and 10-DEBC (AKT) showed no clear phenotypic alteration compared to the control. Interestingly, FAK inhibitors Y11 and PF-573228, and PI3K inhibitor LY-294002 resulted in smaller (Area), more rounded (Roundness) and denser organoids (Density), which displayed markedly decreased invasiveness (MaxApp), compared to control (Figure 6C). These results suggest that these FAK and PI3K inhibitors affect the morphology of both cell compartments, whereas IWP-2, an inhibitor of WNT processing and secretion, primarily affects tumor growth. Taken together, the inhibitors Y11 and PF-573228, which both target the same Y397 phosphorylation site of FAK, exhibit the most specific therapeutic potential *in vitro*, since they simultaneously constrained tumor and stroma growth and invasion as a whole, without prominent cytotoxicity (Figure 6, Supplementary Figure S9).

### Therapeutic potential of FAK inhibition in PrCa is only detected in a tissue-like cell model

**Figure 7:**
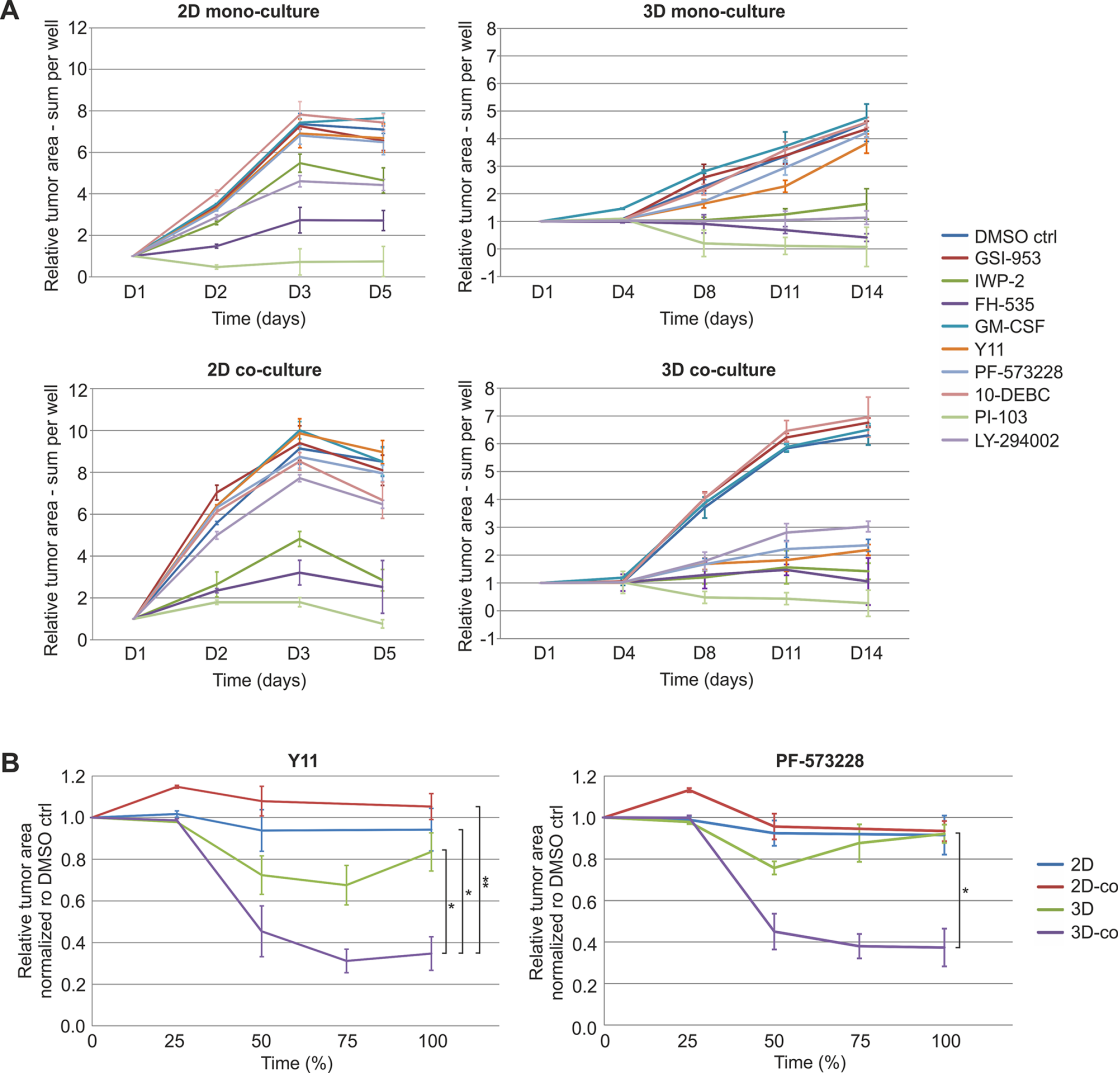
FAK inhibitors significantly reduce tumor growth when relevant tumor microenvironment is included **A.** Comparison of tumor cell/organoid growth in 2D mono- and co-culture as well as 3D mono- and co-culture models treated with small molecule inhibitors. Tumor area (red fluorescence) was segmented and quantified from PE Operetta High Content Imaging System confocal live-cell images (2x) of dsRed-LNCaP and GFP-PF179T cells cultured in different settings and treated with inhibitors (3 μM) or GM-CSF (0.3 ng/ml). Segmentation was performed by the PE Harmony software, to generate comparable growth curves. D: days in culture. **B.** Tumor growth curves for different culture settings treated with FAK inhibitors Y11 and PF-573228. The relative tumor area measures of compound treatments were normalized to DMSO control. To compare the different time frames of the 2D and the 3D experiments, the final time point for all experiments was set to 100%. Error bars depict SEM. *P*-values are * *P* < 0.05, ** *P* < 0.01, by Student *t*-test.

Our platform specifically facilitates the functional evaluation of perturbants on complex 3D co-cultures and tumor microtissues, and is able to identify phenotypic drug effects that are promoted by the stromal counterpart and 3D conditions. To prove this concept, a comparison of growth inhibition between standard 2D mono- and co-cultures, along with 3D mono- and co-cultures, using the same panel of perturbants, was conducted. Quantification of LNCaP cell/organoid area uncovered substantial differences in compound sensitivity and efficacy between the distinct culture settings (Figure 7A, Supplementary Figure S10–S11). We observed significantly reduced tumor organoid area specifically for the two FAK inhibitors (Y11, PF-573228). These effects were most striking in the tissue-like co-culture model that comprises tumor organoids interacting with CAFs (Figure 7B, Supplementary Figure S10–S11). Tumor growth inhibition using FAK inhibitor PF-573328 at day 14 was significantly stronger in 3D co-culture (63% inhibition compared to DMSO control), in contrast to 3D monoculture lacking stromal cells (8% inhibition compared to DMSO control) (Figure 7B). Therefore, both direct and indirect tumor/stroma interactions, targeted by FAK inhibitors, may contribute to the specific effects observed *in vitro*. FAK inhibitor PF-573328 was not more effective in 3D culture (8%) than in 2D co-culture (7%) or 2D mono-culture (9%) settings, all relative to DMSO control (Figure 7B). Our results indicate that significant FAK inhibition of PrCa was only detected in the combination with tumor-stroma and surrounding ECM. These results highlight the importance of the complex 3D co-culture model and the need to include functional CAFs in order to recapitulate relevant biology most completely. On the contrary, the reduction of tumor growth caused by PI3K inhibitor LY-294002 in 2D and 3D monocultures was rescued by addition of fibroblasts (Figure 7A, Supplementary Figure S10–S11). Our approach allows the dual, automated tracking of both tumor and stroma morphology and their intrinsic dynamics. It therefore allows predicting drug sensitivity and efficacy based on representative tumor morphology. This concept should be extremely useful for biologically more informed functional evaluation and chemosensitivity testing of cancer therapeutics.

## DISCUSSION

In the tumor microenvironment, CAFs behave in a fashion similar to active tissue damage and inflammatory responses, and cancer has been described as “a wound that never heals” [42]. CAFs migrate as a cohesive unit via cohort formation and exert a mechanical pressure on the tumor invasion front, capable of changing the dynamics of the entire tumor mass [14] and initiate invasive processes and EMT. CAFs are therefore key determinants for understanding the dynamics, or dynamic behaviour of tumor growth, progression and invasion, and represent an important target for effective cancer therapies. Correspondingly, we observed that both PF179T and WPMY-1 cells formed syncytia while surrounding the tumor organoids, and significantly increased tumor proliferation as well as invasive properties in a cell density-dependent manner (Figure 4D). To form a dynamic cohort, CAFs undergo a cell-to-cell adhesion program to form a syncytium, via formation of adherens- and gap junctions [14]. CAFs can also effectively remodel the ECM. Their motion tracks within the matrix are sufficient to promote cancer cell invasion [8, 31, 32]. Monitoring cancer progression using multiphoton microscopy has to some extent provided insights into CAF migration in ECM [8, 43]. Our 3D co-culture model, to the best of our knowledge, is the only platform that allows simulation and tracking of collective migration of CAFs acting as a cohort, thus leading the way for single tumor cells or even large tumor organoids in collagen containing ECM (Figure 3A–3B, Supplementary Video S1B–S1C). These results illustrate the importance of modeling the CAF dynamics, since fibroblasts can create lasting, pro-invasive changes in the tumor microenvironment, e.g. via cross-linking and re-orientation of collagen fibers, thus generating cancer-associated collagen connected to e.g. tumor stiffness [44, 45]. Detailed, matching analyzes of both the tumor organoid and CAF morphology should therefore be highly informative when validating molecular drug targets for cancer treatments, in particular related to dynamic processes including invasion, motility, and possible metastasis. Our image analysis approach is adaptable to a broad range of tumor and stromal cell types, and does not necessarily depend on the use of labeled cells as morphometric analyzes can also be performed with unlabeled cells. Thus, organotypic co-culture models can be expanded beyond the field of PrCa, and general dynamic changes between tumor and stroma can be quantified by utilizing standard live-cell imaging methods in combination with our technology. Our complex co-culture models are suitable for measuring morphometric effects of anti-cancer drugs on either one of the compartments, thus enabling highly informative and biologically relevant, phenotypic compound screens.

Real-time growth and motion analyzes from low-resolution time-lapse images were in agreement with in-depth phenotypic analyzes from high-resolution confocal images, although both approaches exhibited distinct properties. To demonstrate the usefulness of our method, microtissues were treated with compounds that perturb critical functions in tumor pathobiology. We found that FAK-signaling, which involves direct cell-cell interactions [39], was particularly important for the tumor-stroma crosstalk in PrCa (Figure 5, 6). FAK expression is upregulated in many tumor types including colon, breast, prostate, and pancreas, correlating with poor patient survival [46, 47]. Several clinical trials have been initiated with FAK inhibitors over the past years [39], but so far no drugs have been approved for cancer chemotherapy. The role of FAK-signaling in stroma was first introduced in an orthotopic mouse model of pancreatic cancer, where inhibition of FAK reduces the growth, invasion and metastasis, concomitant with lowered number of tumor-associated macrophages and fibroblasts [39, 47]. Inhibition of FAK in our PrCa 3D co-culture model showed a similar phenotype, i.e. resulting in smaller organoids and decreased invasiveness (Figure 6), combined with reduced size, mobility and branching of CAF cohorts (Figure 5). In contrast, no evident phenotypic changes were detectable in conventional 2D co-cultures (Figure 7). Our results indicate that in addition to promoting tumor progression of PrCa cells, FAK induces CAF cohort spreading in the surrounding tumor microenvironment. Specific functional effects detected with FAK inhibitors (Y11, PF-573328) showed significantly less pronounced effects on tumor cells in the absence of fibroblasts, compared to microtissues in which CAFs were present. The effects could be due, at least in part, to blocking direct cell-cell-interactions between tumor cells, as well as between tumor and stromal cells. This finding highlights the importance of the stromal component, which can simultaneously interact via both paracrine and secretory pathways with tumor cells. In conclusion, blocking of cell-cell-interactions between tumor and stroma should be considered as an important additional cancer target.

We demonstrate that our computational analysis approach can be utilized for meaningful quantitative morphometric measurements of 3D co-cultures. The experimental platform enables functional evaluation of phenotypic responses to anti-cancer drugs, and allows the simultaneous tracking of highly dynamic and heterogeneous tumor-stroma interactions. Our results reveal the potential of standardized and miniaturized tissue-based model systems as valuable tools for phenotypic, high-content screening, in particular when it is combined with matching automated image analysis systems. Our biologically relevant and complex model system, which recapitulate the tumor architecture, histology, and heterogeneity more faithfully, may also reduce, refine and replace the need for mouse experiments in early stage drug discovery. These microtissue-based model systems would significantly facilitate target validation related to the tumor microenvironment and invasion.

## MATERIALS AND METHODS

### Cell lines and culture conditions

All cell lines were obtained from American Type Culture Collection (ATCC) or originator laboratories. PrCa lines were propagated in RPMI-1640 (Sigma-Aldrich), CAF lines PF179T and WPMY-1 were cultured in DMEM (Sigma-Aldrich), and both media were supplemented with 10% FBS, 1% penicillin/streptomycin and 1% L-glutamine. Immortalized, non-transformed epithelial cell lines EP156T cells were cultured in Keratinocyte Serum-Free Medium (KSFM), supplemented with 50 mg/l bovine pituitary extract, 5 μg/l EGF and 2% FBS for 3D conditions. 1 nM of the synthetic androgen R1881 was added to VCaP medium for growth support.

### Miniaturized 3D cultures in ECM

All 3D experiments shown were performed in 96-well angiogenesis plates (Ibidi), exhibiting a well-in-a-well geometry that consists of two compartments, the smaller residing within the bottom of the larger well. This geometry reduces the meniscus formation caused by liquid tension, results in an even surface supporting rapid, automated microscopic imaging and autofocusing. The method further allows cells to be embedded in a defined and narrow focal plane between two layers of ECM (sandwich system). The volume of a single 3D experiment requires only 30 μl of ECM: growth factor-reduced Matrigel (Corning), stock 8 mg/ml and collagen type-I (Corning), stock 3 mg/ml, or a 1:1 mixture of both. The pH of collagen type-I was neutralized with NaOH prior to use. Miniaturized 3D co-cultures were prepared as described previously [2, 27]. Bottom wells of Ibidi Angiogenesis μ-plates were filled with 10 μl of 50% ECM in medium, and incubated at +37°C for 30–60 min. The plates were then centrifuged for 20 min. at 200 × g. Single cell suspensions were mixed with 25% ECM in medium and placed on top of the polymerized bottom gel at a density of 700–1500 cells/well, depending on the cell line. Hence, one tumor organoid is typically generated from a single cell (clonal approach). The plates were centrifuged for 10 min. at 100 × g, and the upper gel was allowed to polymerize at +37°C for 3–4 h or overnight. The wells were then filled with medium, containing 2% FBS, 1% penicillin/streptomycin and 1% L-glutamine, and medium was changed every 3^rd^ – 4^th^ day. The μ-plates were humidified by adding sterile PBS into the outer wells and rim.

### Optimization of 3D co-culture conditions

In order to generate stable and reproducible 3D co-cultures, the culture conditions for every cell line had to be optimized: cell ratios, FBS concentration and ECM composition. Regarding dsRed-LNCaP tumor cells in combination with GFP-PF179T fibroblasts [28], we observed that a ratio of 1:2 in favor of the tumor cells generated the most stable and tissues-like culture. Addition of larger numbers of fibroblasts caused contraction of the culture and the matrix, whereas fewer fibroblasts did not succeed to form a proper stromal network surrounding the organoids. Regarding dsRed-LNCaP tumor cells in combination with GFP-WPMY-1 fibroblasts [36], we detected that a ratio of 1:10 in favor of the tumor cells generated the most stable and tissues-like culture, after 8–11 days in 3D culture. The microtissues then remain virtually unchanged for an additional 1–2 weeks after homeostasis has been established. To examine the added value of stromal cells and factors secreted by them, low serum conditions, i.e. 2% FBS were consistently applied to all 3D cultures. We also noticed that 10% FBS inhibited the growth of the tumor organoids when co-cultured with PF179T cells (data not shown), likely due to additional growth factor secretion by the fibroblasts. Marked alterations in fibroblast dynamics and tumor organoid morphology were found when CAFs were co-cultured with LNCaP cells in different matrices. In summary, a mixture of Matrigel (laminin) and collagen type-I resulted in the most ideal growth conditions, favoring both tumor and stromal cells, respectively.

### Pre-formed spheroids in low-attachment culture

VCaP cells were alive but failed to grow when directly embedded as single cells into any ECM formulation. To facilitate the growth of VCaP cells in ECM, we pre-cultured floating cell aggregates, typically loosely adhered spheroids, in low-attachment culture (bacterial Petri dish). After 3–5 days with regular suspension of the cells, the VCaP cells started to attach to each other, and small, first irregular and later increasingly round spheroids were formed. These pre-formed spheroids were then transferred into ECM and gave rise to proliferating organoids. 1 nM synthetic androgen R1881 was added to VCaP medium for growth support.

### Real-time live-cell imaging using incucyte FLR

Live-cell imaging was carried out in a systematic, standardized fashion. Incucyte FLR live-cell fluorescent imaging device (Essen Bioscience) was utilized for continuous monitoring of up to four weeks of 3D culture. Settings were selected to capture one image per well/per hour. These image series are based on phase contrast microscopy combined with green fluorescence, generating image sequences that can be visualized as videos, ideal for investigations of long dose-response and time-course experiments in 3D co-cultures. The cultures were also stained with CellPlayer™ Kinetic Caspase-3/7 Apoptosis Assay Reagent (Essen Bioscience), diluted 1:1000 in culture medium for live-cell imaging of apoptosis. The reagent was mixed in the cell culture medium and pipetted into wells, incubated at +37°C for 72 h, and monitored in real time with the IncuCyte FLR.

### Stabilization of real-time live-cell image videos

The time-lapse movies displayed shaky motions. Sequential image capturing used in the process of recording cellular motility introduced visible shaking of the time-lapse movies. The changes in camera positioning create differences in the field of view and focus. The variation in image position was corrected by using stabilized frame-by-frame alignment since the subsequent analyzes require steady, positioned time-lapses. Furthermore, video stabilization was also necessary to enable continuous visualization of the 3D co-cultures over time, without distorting camera movements. Stabilization was performed in two steps: global motion estimation and motion compensation. A translation motion model was adopted to estimate suitable values based on the shift property of the Fourier Transform using phase correlation [48]. Given two images *f* and *g*, which are related by a simple translation (Δ), then the corresponding Fourier Transforms are denoted as *F*(*u*) and *G*(*u*) (i.e. *G*(u) = e^−2πiu·Δ^*F*(u)). Then the normalized cross-power spectrum (phase correlation) is:

$$T\left(u\right)\equiv \frac{F.G^{*}}{\left|F\right|.\left|G\right|}\left(u\right)=e^{2\pi iu.\Delta }$$

Under these premises, the Inverse Fourier Transform results in a single peak, which the location of the peak corresponds to the motion vectors between the two frames. For heavily blurred movies, we utilized an improved version of the original phase correlation method [49], which is more robust in registering blurred images. During the motion compensation step, maximum overlap among the frames was calculated and the point of view was fixed by shifting frame centers according to estimated motion vectors in the previous step. Therefore, frames in stabilized videos were cropped.

### Filtering of real-time live-cell image videos

The fluorescent images tended to blur, and show noise and high background. A lack of proper focus, and auto-fluorescence were the main sources of these errors. In this study, cascaded filters were utilized to suppress the effects of the aforementioned problems in further image analysis steps. Median filtering and non-local means de-noising [50] were applied, respectively. The median filter is a non-linear filter and was preferentially used to reduce spikes and “salt-and-pepper noise” (impulsive noise). We utilized a window of 5 × 5 pixels. Finally, a non-local means algorithm, which depends on self-similarity concept, was employed with a patch size = 7 × 7 pixels, a search window size of = 31 × 31 pixels, and a weight decay control parameter depending on the standard deviation of the noise *h* = 15. However, these default parameters can be tuned manually for adaptation to divergent image sequences.

### Segmentation and growth analysis of fibroblasts

Cell segmentation is an essential step in quantifying fibroblast cell dynamics. Adaptive thresholding was applied to the green channel to segment fluorescent images. Due to the granular nature of the ECM, auto-fluorescence, lack of proper focus and high background, the fibroblasts exhibited low contrast and intensity-variant characteristics as a function of time. Therefore, selecting a single global intensity threshold based on the histogram such as the widely used Otsu threshold resulted in poor segmentation of cells from background. To overcome these problems, we employed local adaptive thresholding [26], so that the threshold value *T*(*x, y*) is calculated individually for each pixel *p*(*x, y*). The threshold value is then determined using a square block (width = height) neighborhood of *p*(*x, y*), which cross-correlated with a Gaussian window of the block size minus a constant κ:

$$T(x,y)=(\sum_{i=-j}^{k}\sum_{j=-k}^{k}f(x-i,y-j)w(i,j))-\kappa$$

where

$$w\left(i,j\right)=\frac{1}{2\pi \sigma^{2}}e^{-\frac{i^{2}+j^{2}}{\sigma^{2}}}$$

is a (2*k* + 1) × (2*k* + 1) Gaussian kernel. After binarization, contour detection was used to obtain the boundaries of cell clusters. Due to the highly complex nature of 3D cultures, it was not feasible to detect individual cells from fluorescent images. Therefore, we focused on detecting and quantifying multicellular CAF cohorts instead. In the final step of the segmentation, small noisy regions were removed. We associated fibroblast growth (area measured in pixels) with the size of the segmented region (i.e. total number of pixels in the fluorescent image covered by fibroblasts). Although volumetric information gave more accurate results for cells cultured in 3D matrix, area information provided a reasonable estimation to compare different conditions or treatments.

### Motion analysis of fibroblasts

Cell motility (motion) was assessed by using the dense optical flow algorithm. Previous motion analyzes efforts have mainly aimed at tracking high intensity regions (detections of circular cells/nuclei) in image sequences. Therefore, tracking performance is heavily based on the detection success (precision-recall). High contrast, sufficient and consistent intensity differences between cell and its neighboring, and low noise levels are essential for successful automation. The fluorescent co-culture image sequences presented in this study had a problem with noise and area definition. Furthermore, individual cell tracking for motion analysis was not convenient since such approaches result in high errors with large variances among different image sequences. Instead, the dense optical flow of video sequences that is independent of cell detections provided better estimates for the cell movement analysis. The goal of optical flow estimation is to compute an approximation to the motion field from time-varying image intensity. Optical flow is a rich source of information that supports understanding of cell motility. We employed the Farnebäck algorithm [29], to estimate dense optical flow and to assess where fibroblast cohorts have moved in the subsequent frame. The dense optical flow measure proposes to approximate the neighborhood of each pixel with a quadric polynomial, and then analyze what occurs if a polynomial undergoes a translation. Optical flow was determined by searching locations where a surface (represented by a quadric polynomial), has moved between two subsequent frames. The local region was represented by a 3D surface:

$$f_{1}\left(x\right)=x^{T}A_{1}x+b_{1}^{T}x+c_{1}$$

where A was a symmetric matrix, b a vector and c a scalar. A new signal *f_2_* was then constructed by a global displacement d:

$$f_{2}\left(x\right)=f_{1}\left(x-d\right)={\left(x-d\right)}^{T}A_{1}\left(x-d\right)+b_{1}^{T}\left(x-d\right)+c_{1}=x^{T}A_{2}x+b_{2}^{T}x+c_{2}$$

Equating the coefficients in the polynomials resulted in a solution for the translation vector d:

$$A_{2}=A_{1}$$

$$\;b_{2}=b_{1}-2A_{1}d$$

$$\;c_{2}=d^{\text{T}}A_{1}d-b_{1}^{\text{T}}d+c_{1}$$

$$\;\;∴d=-\frac{1}{2}A_{1}^{-1}\left(b_{2}-b_{1}\right)$$

After a series of refinements, this method led to a robust algorithm. Finally, we obtained displacement vectors for each pixel in every frame. Subsequently, the mobility of the cell culture objects contained in a single frame was described by the displacement vector magnitudes.

### Shape analysis for evaluation of fibroblast branching

Automated analysis of cell morphology is important in order to understand the functional relationship between altered cell shape and variable cell culture conditions. In this context, we analyzed the branching characteristics of fibroblast cell clusters over time in a quantitative manner. We started with the binary images, resulting from the segmentation processes described above. The shape of each cell cluster formed by CAFs was then analyzed individually. A closed contour of the cell cluster was extracted from the binary image. The convex hull of the contour was detected, and it was defined as the smallest convex shape that contained the contour points. This means that for any two points belonging to the same shape, the whole connecting line segment is also a part of the shape. Contour defects were detected and defined as the area situated between the contour and the convex hull. The property of convexity of the original contour was violated due to these defects, since the contour tended to bent away from the convex hull. The starting and end points of the defect, which forms the line of the defect in the convex hull, were then detected. Finally, the farthest contour point from the line and the distance between the farthest contour point and the hull were determined to account for the cell extension (branch) size. We associated the number of defects with the number of branches in a cluster. Only the defects showing depths (calculated from the farthest point of the convex hull for each defect individually) greater than a certain pre-defined threshold were considered as reliable extensions, in order to reduce noisy measurements.

### Morphometric analysis of fibroblasts

To explore various cell dynamics, experiments were diversified based on the results of segmentation, motion and shape analysis. We fitted the curves resulting from the measurement of image data for visibility reasons, in order to remove noisy measurements. The growth rate was defined as

$$\frac{\text{current fibroblast area}\;(\text{in pixels})}{\text{initial fibrolast area}\;(\text{in pixels})}.$$

The initial fibroblast area normalization was necessary to result in standardized comparisons. After the stabilization step, the field of view tends to differ between image sequences. Similarly, the initial number of cells may also vary. The growth rate of fibroblasts was shown as a function of time. The motility of the CAF cohort in a single frame was described by the magnitude of the displacement vector. The mean motion per frame was defined as the sum of all displacement magnitudes, divided by the sum and the number of image pixels, over time. Displacement vectors for each pixel were obtained in the dense optical flow analysis stage. A segmentation mask was utilized to suppress background regions. The number of extensions per cluster was extracted and mean values were plotted as a function over time. Extension sizes were extracted for branching clusters and mean values were evaluated separately for each cluster. Finally, the mean of these values per frame were plotted over time.

### Segmentation and growth analysis of tumor organoids

Intensive de-noising was applied for tumor segmentation due to low signal-to-noise ratio and high background clutter; a two cascaded non-local means denoising followed by means shift filtering [51]. Similar to fibroblast analysis, adaptive thresholding was applied and the segmentation was smoothed using a morphological filter. The segmentation also detected fibroblast cohorts, which had to be removed. As fluorescent and phase contrast images are registered, we masked the phase contrast segmentation result with the fibroblast segmentation, obtained from fluorescent images. In this tumor growth analysis, identical fixed parameters were utilized for all sequences. The image segmentation of cells growing in pure collagen did not work optimally due to noise and image granularity. Instead, we focused on using a mixture of Matrigel/collagen, where the segmentation process worked consistently and results were reliable.

### Confocal image acquisition, pre-processing

3D confocal images were acquired with a Zeiss Axiovert-200M microscope, equipped with Yokogawa CSU22 spinning disc confocal unit using Zeiss Plan-Neofluar 5× or 20× objectives. Maximum intensity projections were created with SlideBook (Intelligent Imaging Innovations Inc.). Background noise was removed by image normalization, also using SlideBook. Confocal microscopy settings of Leica TCS SP5 microscope were used for second-harmonic generation microscopy of collagen fibers, using a 40× objective. Maximum intensity projections and image normalizations were done with LAS AF Lite (Leica Microsystems).

### Immunofluorescence of 3D co-cultures

The 3D co-cultures were fixed in the μ-angiogenesis wells with 2% paraformaldehyde (PFA) in PBS for 20 min, washed with cold PBS and blocked in 20% horse serum. Cell permeabilization was performed by addition of 0.7% Triton X-100 (Sigma-Aldrich). Fixed cultures were incubated at 4°C overnight with primary antibodies (1:100), washed with PBS and incubated for 3–4 h, at RT with secondary antibodies (1:500) and Draq5 (1:1000, Cell Signaling Technology), to visualize the nuclei and AlexaFluor^®^ 488 Phalloidin (1:50, Life Technologies), to detect F-actin. The following primary antibodies were used: anti-vimentin (SP20) and anti-E-cadherin (HECD-1, both from Abcam), and anti-laminin-α1 (LAM-89, Santa Cruz). Images were taken with the Zeiss Axiovert-200M spinning disc confocal microscope, using a 20x objective. Z-stacks were acquired with a step-size of 8 μm. The images were pre-processed using SlideBook and NIH Image J.

### Transmission electron microscopy (TEM)

Samples were fixed in 1% glutaraldehyde and 4% formaldehyde in 0.1 M phosphate buffer, pelleted, immersed in 2% agarose in distilled water and post-fixed in 1% osmiumtetroxide, dehydrated in acetone and embedded in Epon LX 112 (Ladd Research Industries). Thin sections were cut with Leica Ultracut UCT ultramicrotome, stained in uranyl acetate and lead citrate, and examined in Tecnai Spirit transmission electron microscope. Images were captured by a Quemesa CCD camera (Olympus Soft Imaging Solutions GMBH).

### Morphometric analysis of tumor organoids

Tumor organoids in maximum intensity projections were segmented using the AMIDA software [27]. In summary, AMIDA is a multi-parametric image analysis program designed for high-content analysis of complex and heterogeneous 3D organoid cultures. The software first identifies individual multicellular structures by image segmentation, and assigns numerical values for selected cancer-relevant parameters to the objects; these are then exported as a text file (csv; comma-separated values). AMIDA is primarily designed to retrieve information from 3D confocal image stacks, and it allows quantitative measurements of large numbers of images and structures, with a multitude of different organoid shapes, sizes, and textures, see Supplementary Table S1. AMIDA supports automated workflows, and can be combined with quality control and statistical tools for data interpretation and visualization.

### Confocal data annotation and quality control

Raw numerical data were statistically processed and visualized with R/Bioconductor. Statistical analysis and plotting tools implemented for processing numerical data (post-image analysis) were written using R, an open source programming language and software environment for statistical computing and graphics (http://cran.r-project.org). All R-scripts were incorporated in REX, an in-house html-software environment that includes a browser-based user interface.

### Segmentation and analysis of 3D confocal image stacks

Segmentation of the 3D confocal image stacks was achieved using a Markov random field based methodology [52]. For every pixel i in an image, let *x^i^* and *z^i^* be the unobserved pixel label and observed pixel intensity, respectively. Given the observed pixel intensities *z*, we found the labelling (segmentation) $x^{*}$ that minimizes the energy function

$$E(x)=\sum_{i}\sum_{l}\theta i;lI\{xi\;=l\}+\sum_{\left(i,j\right)}\sum_{l}\sum_{k}\theta ij;lkI\{xi=l,xj=k\}$$

for pixels *i* and adjacent pairs of pixels (*i*, *j*) with labels *l* and *k*. The unary potentials were set to be

θi;l = − log(fl(zi))

where *fl* is the density of the pixel values associated with label l. The pairwise potentials were set to be

$$\theta ij;lk\;=\{\begin{array}{cc} \lambda_{0}+\lambda_{1}\;exp\left\{\frac{-1}{2\beta }{\left||z_{i}-z_{j}|\right|}^{2}\right\} & if\;l\neq k\\ 0 & if\;l=k. \end{array}$$

The raw images were first scaled to 8-bit representations and processed using a local entropy filter. A mixture model with three components was fit to the density of the pixel values. The mixture components were used to set the unary potentials of the three labels corresponding to ‘in focus cells’, ‘cell shadow’ and ‘background’. The pairwise potential parameters λ0, λ1 and β were manually set based on the distribution of the unary potentials. The segmentation methodology was implemented in MATLAB using the α-expansion algorithm to find the minimum energy labelling [53–55]. The segmentation was performed separately for the different channels corresponding to ‘LNCaP organoid’ and ‘PF179T CAF’. In the segmentation output, the pixel labels for ‘LNCaP organoid’ and ‘PF179T CAF’ have been overlaid to display co-localization.

### Compound treatments

All compounds were purchased from Tocris Bioscience (Table 1), and dissolved in DMSO. The panel of inhibitors was selected from pathways known to interfere with cell-cell contacts in tumorigenesis. Multiple growth factors were also tested in 3D culture (data not shown), of which GM-CSF was selected to be included in the 3D co-culture experiments. All exposures were performed in triplicates, including vehicle control. Compound treatments were initiated after 4 days of 3D culture, when significant organoids had formed, and continued for an additional 10 days. Final concentrations used in the experiments: inhibitors 3.0 μM; GM-CSF 0.3 ng/ml.

### PE Operetta and harmony single fibroblast analysis

3D confocal images were acquired with the PE Operetta High Content Imaging System, using 2x or 10x objectives. Maximum intensity projections were created with PE Harmony High Content Imaging and Analysis Software. This software was used for analysis of the high-content imaging data, including the usage of existing image analysis building blocks, for creating high content image analysis applications tailored to fit both tumor organoid and single fibroblast analyzes.

### Comparison of 2D mono- and co-cultures with 3D mono- and co-cultures

Confocal images of the whole well were acquired with PE Operetta High Content Imaging System, using 2x objective. Maximum intensity projections were created with PE Harmony (High Content Imaging and Analysis Software). This software was used for segmentation and measurement of the red fluorescent tumor cell/organoid area (sum per well, μm^2^), both from 2D and 3D cultures, to generate growth curves. To compare the different time frames of the 2D and the 3D experiments, the final time point for all experiments was set to 100%, and the relative tumor area measurements were normalized to DMSO control.

## SUPPLEMENTARY FIGURES, VIDEOS AND TABLE




























