# Supplementary material for: Automated tracking of tumor-stroma morphology in microtissues identifies functional targets within the tumor microenvironment for therapeutic intervention
Source: Oncotarget. 2015 Sep 3;6(30):30035–56. doi: 10.18632/oncotarget.5046 (PMC4745780; doi:10.18632/oncotarget.5046)
Supplement: Supplementary file 1 [file oncotarget-06-30035-s001.pdf]

SUPPLEMENTARY FIGURES, VIDEOS AND TABLE

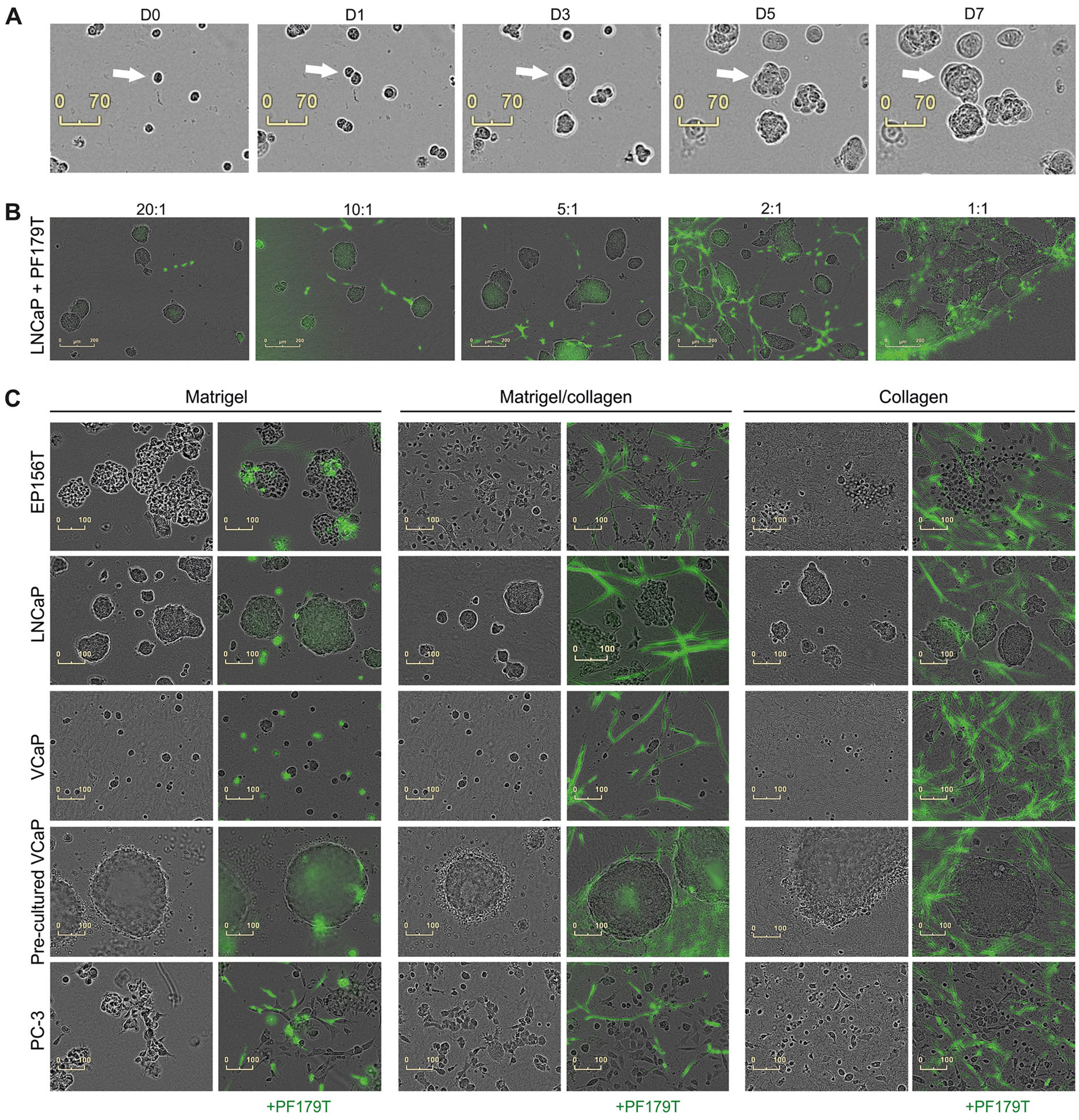

Supplementary Figure S1: Establishment of 3D co-cultures from several PrCa cell lines together with PF179T CAFs cultured in different matrices. A. Incucyte image sequence displaying that tumor organoids are generated from single LNCaP cells when cultured in Matrigel/collagen matrix. Arrow depicts a single cell forming an multicellular organoid over 7 days. D: Day. **B.** Optimization of initial cell seeding ratios. LNCaP and PF179T cells were cultured in Matrigel/collagen mixture and representative live-cell images are shown. A ratio of 2:1 in favor of the tumor cells was optimal, assuring sufficient numbers of fibroblasts that did not lead to contraction of the matrix. Scale bar: 200 μm. **C.** Real-time live-cell imaging of EP156T, LNCaP, VCaP, VCaP pre-cultured organoids and PC-3 (phase contrast) and PF179T CAFs (green) co-cultured for 8 days in Matrigel, collagen and mixture. LNCaP and VCaP pre-cultured spheroids formed structures that most faithfully recapitulated the histology of PrCa tissue *in vivo*. Initial cell seeding ratios were the same in all 3D co-cultures; epithelial cells: PF179T CAFs 2:1, and cells were cultured in 2% FBS. Scale bar: 100 μm. (*Continued*)

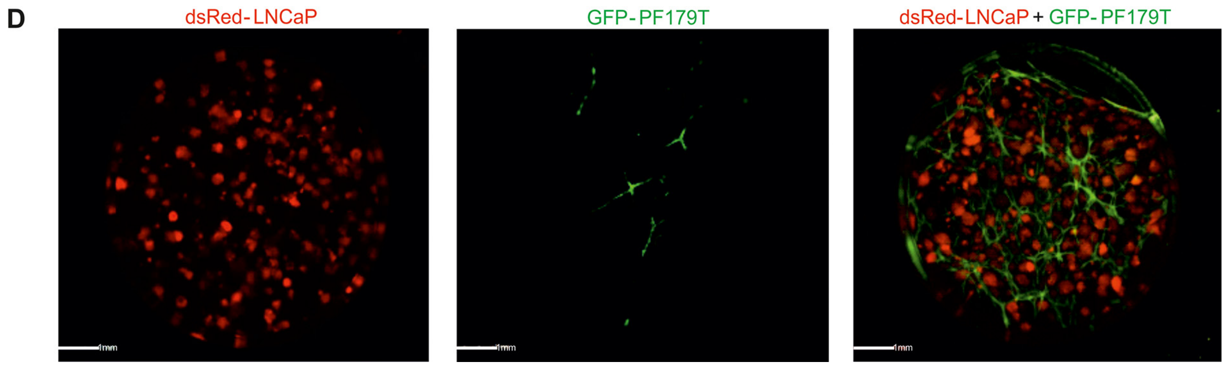

Supplementary Figure S1: (*Continued*) Establishment of 3D co-cultures from several PrCa cell lines together with PF179T CAFs cultured in different matrices. D. Example confocal images (2x) that capture the entire well, specifically showing that even in appropriate ECM, fibroblasts grow poorly in absence of tumor cells, indicating that CAF cohorts require the cross-talk with cancer cells for optimal growth, motility and and generation of branches. Left panel: LNCaP monoculture, middle panel: PF179T monoculture, right panel: LNCaP+PF179T co-culture. Scale bar: 1 mm.

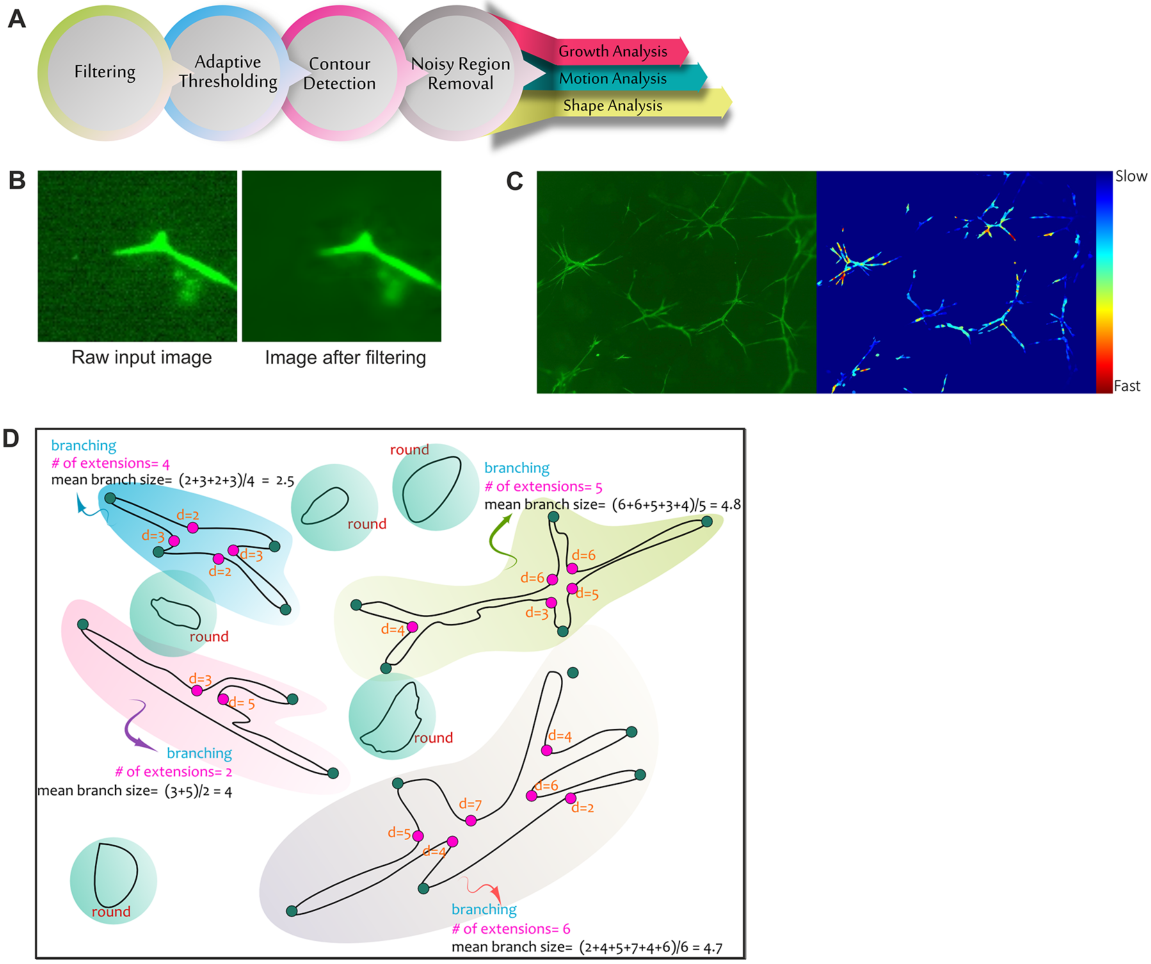

Supplementary Figure S2: Automated analysis of fibroblast and tumor dynamics in Incucyte FLR time-lapse image sequences. A. The image analysis approach is described as a flowchart. **B.** Median filtering and non-local means de-noising was applied. Example images include raw and filtered images of GFP-PF179T CAFs. **C.** Fibroblast dynamics was analyzed from Incucyte FLR time-lapse image series in an automated fashion. An example image for the segmentation used in optical flow analysis is shown. Relative fibroblast motility is encoded by the color map (right panel). **D.** Description of the various morphometric measurements used for statistical analysis of fibroblast branching. d: distance. (*Continued*)

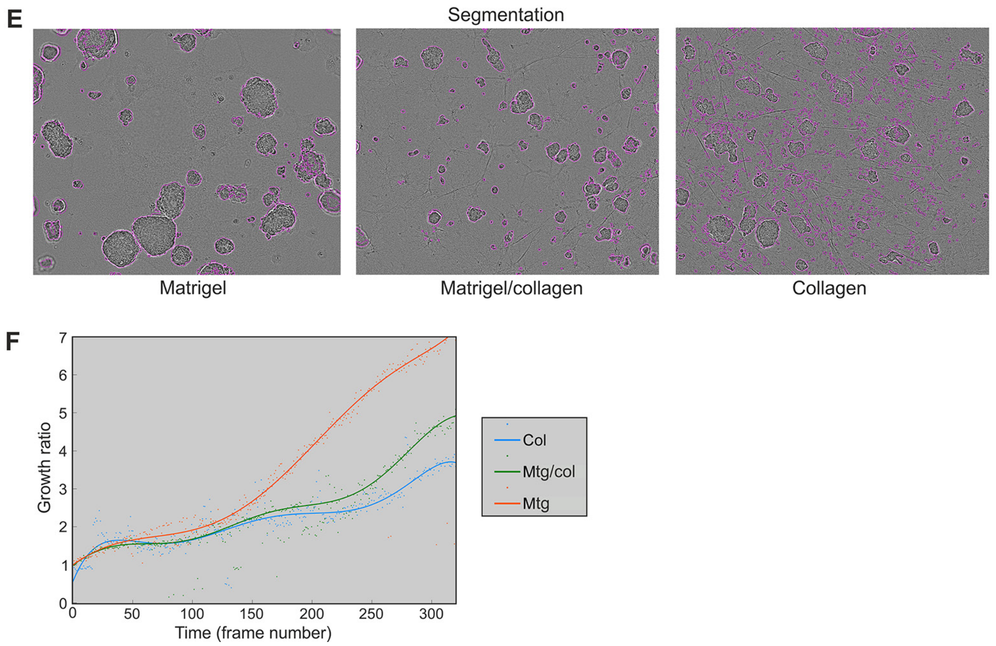

Supplementary Figure S2: (*Continued*) Automated analysis of fibroblast and tumor dynamics in Incucyte FLR time-lapse image sequences. E. Analysis of LNCaP growth in co-culture with PF179T cells. Example phase contrast images illustrate the segmentation of organoids in Matrigel, Matrigel/collagen mixture and collagen. Segmentation of tumor organoids is shown in violet. The image segmentation of organoids in pure collagen did not work optimally due to background noise. **F.** Quantification of tumor organoid growth rate (area) from real-time live-cell image sequences is shown over time. Every dot represents a value per each time frame of the image sequence.

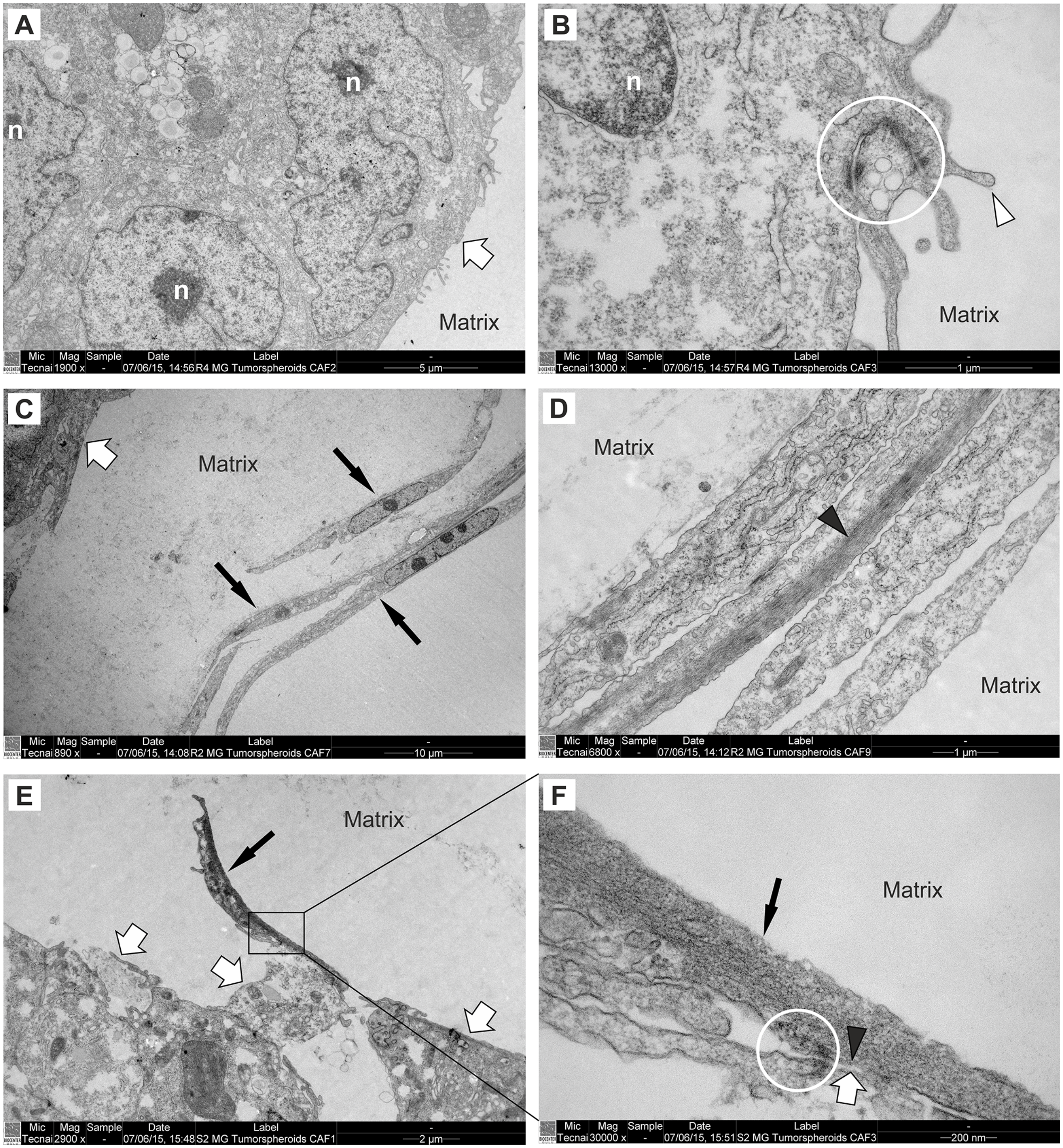

Supplementary Figure S3: Direct contact between CAFs and tumor organoids was visualized using transmission electron microscopy. A. Part of a tumor organoid. Scale bar: 5 µm. **B.** Tumor surface of an organoid in higher magnification. Tumor cell-cell contact, indicated by cortical actin deposition, is encircled. The tumor cell shows small invasive protrusions. Scale bar: 1 µm. **C.** Tumor organoid in close proximity to CAFs. The distance between tumor organoid and CAFs is approximately 20 µm. Scale bar: 10 µm. **D.** CAF cells in a higher magnification. Scale bar: 1 µm. **E.** A CAF cell in direct contact to a tumor organoid. Scale bar: 2 µm. **F.** Marked area in panel E is blown up, showing the α-smooth muscle actin (α-SMA) positive CAF plasma membrane, which is very closely located to tumor cell membrane (distance only a few nm). Circle highlights electron dense material, indicating cortical actin accumulation, a hallmark of direct cell-cell contacts (also seen in panel B). Scale bar: 200 nm. White thick arrow: tumor organoid, black thin arrow: α-SMA positive CAF, n: nucleus, matrix: matrix or empty space (due to sample preparation) surrounding the organoid, white arrow head: invasive protrusion, black arrowhead: α-SMA-rich cytoskeleton.

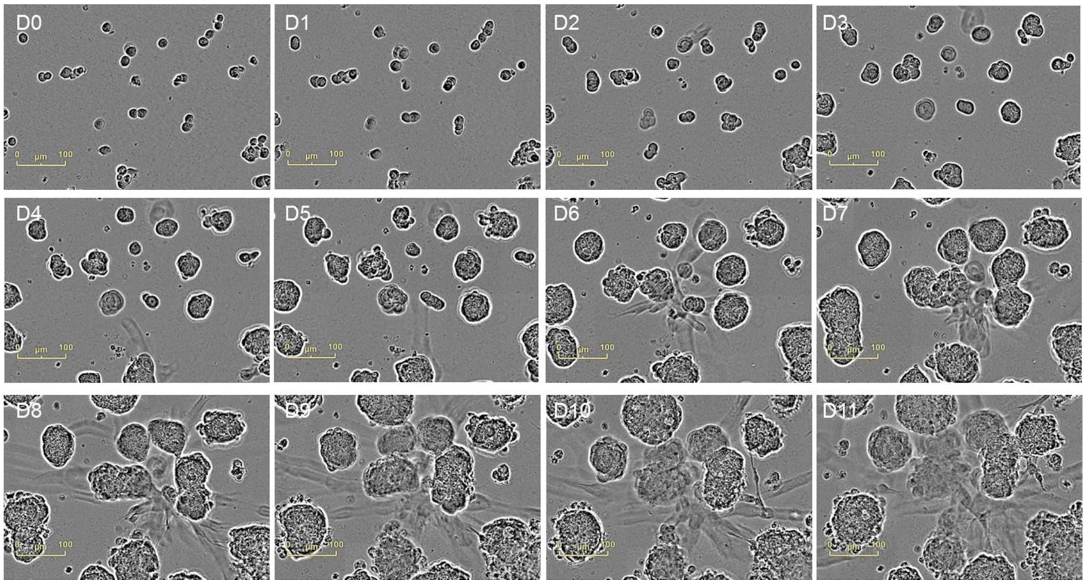

Supplementary Figure S4: WPMY-1 CAFs in 3D co-culture with LNCaP cells require only a minute amount of CAFs. A ratio of 1:10, in favor of the tumor cells was sufficient to generate a structured microtissue after 14 days. 4 days after seeding in Matrigel, WPMY-1 myofibroblasts form a "nucleation" center from which fibroblasts rapidly radiate into the ECM although it lacks collagen, and start to attach to growing organoids. At days 8–11, fibroblasts and tumor organoids together form stable co-cultures that barely change over time. Scale bar: 100 µm.

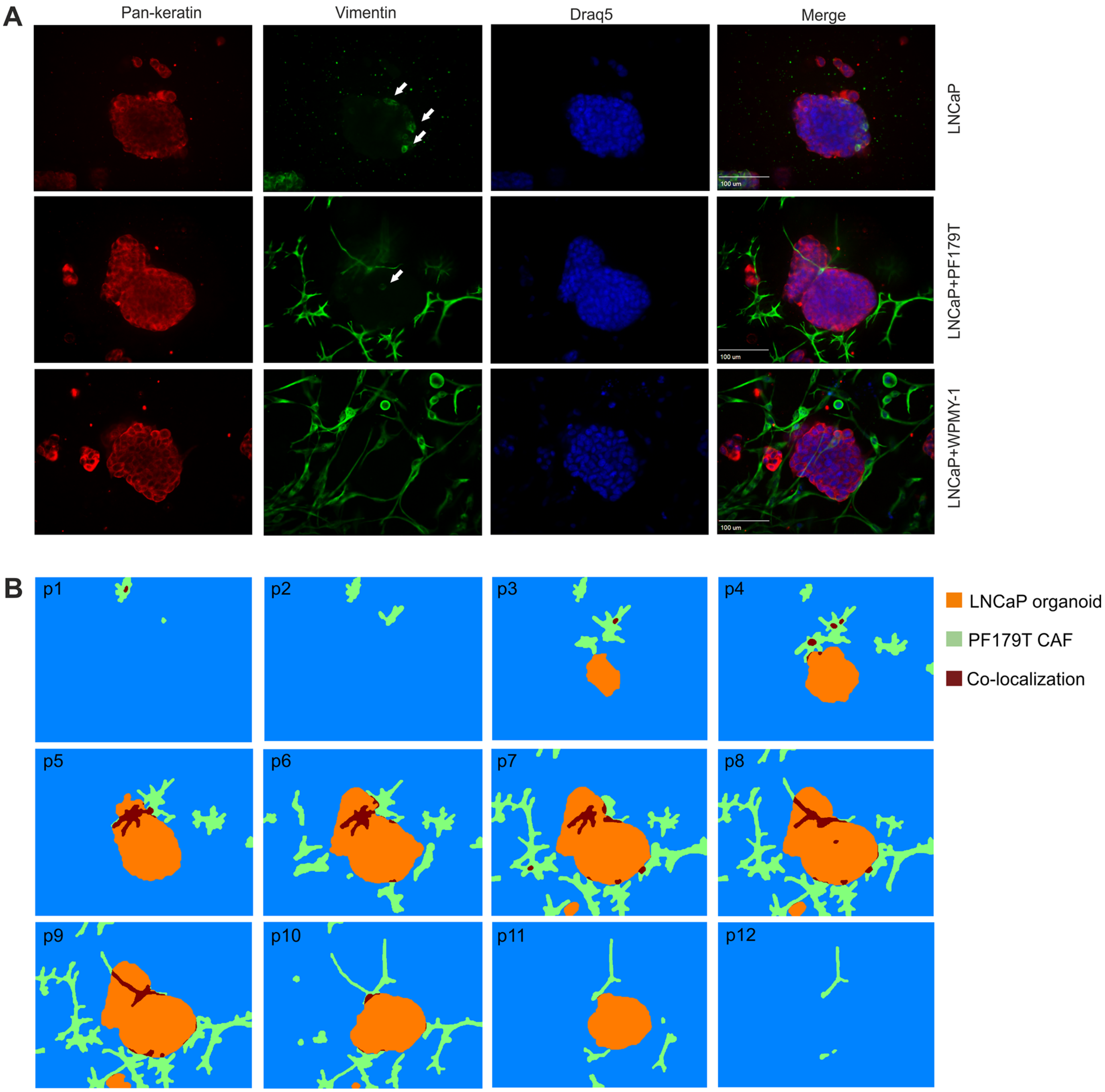

Supplementary Figure S5: PF179T CAFs surround and make contact with tumor organoids in microtissues. Stainings and analysis were performed after 12 days of LNCaP cells in 3D mono- or co-culture with fibroblasts (PF179T and WPMY-1). **A.** Fibroblast and tissue morphology generated by PF179T and WPMY-1 markedly differed from each other. IF-stainings including: Pan-keratin (red) expression as a marker for tumor cells of epithelial origin, vimentin (green) as a fibroblast marker, indicating that some of the epithelial tumor cells have partially acquired a mesenchymal phenotype (white arrows), draq5 (blue) as a nuclear counter-stain. Vimentin staining also shows that PF179T cells interact with tumor organoids, whereas WPMY-1 cells surround the organoids. Representative organoids are shown in the figure. Scale bar: 100 µm. **B.** To identify the interaction points between tumor and stroma, which was observed in (A), LNCaP organoids (orange) and PF179T CAFs (green), all the planes of a 3D confocal image stack were segmented. The overlay depicts areas of co-localization (brown), indicating that the CAFs surround the organoids, but also make connections with the tumor organoids via filopodia-like structures. p: confocal planes 1–12.

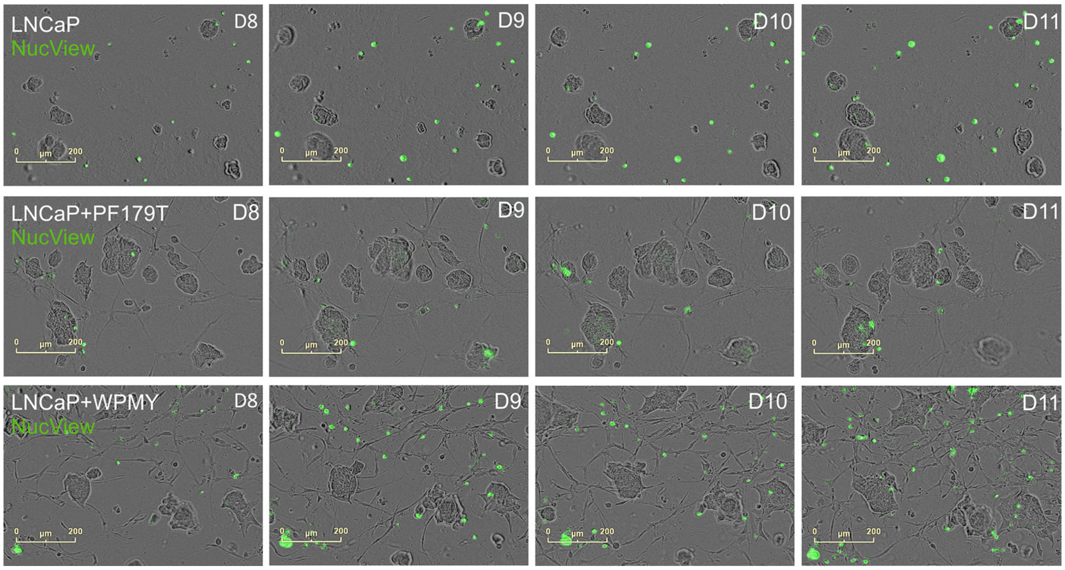

Supplementary Figure S6: Assessment of apoptosis in microtissues. A caspase-3/7 live-cell stain NucView (green) was added to LNCaP cells alone or in 3D co-culture with fibroblasts (PF179T or WPMY-1), after 8 days of culture and followed in Incucyte FLR over 72 h. Addition of fibroblast and generation of microtissues did not result in any detectable increase in apoptosis. Only single tumor cells not incorporated into larger organoids were occasionally undergoing apoptosis. Scale bar 200: µm.

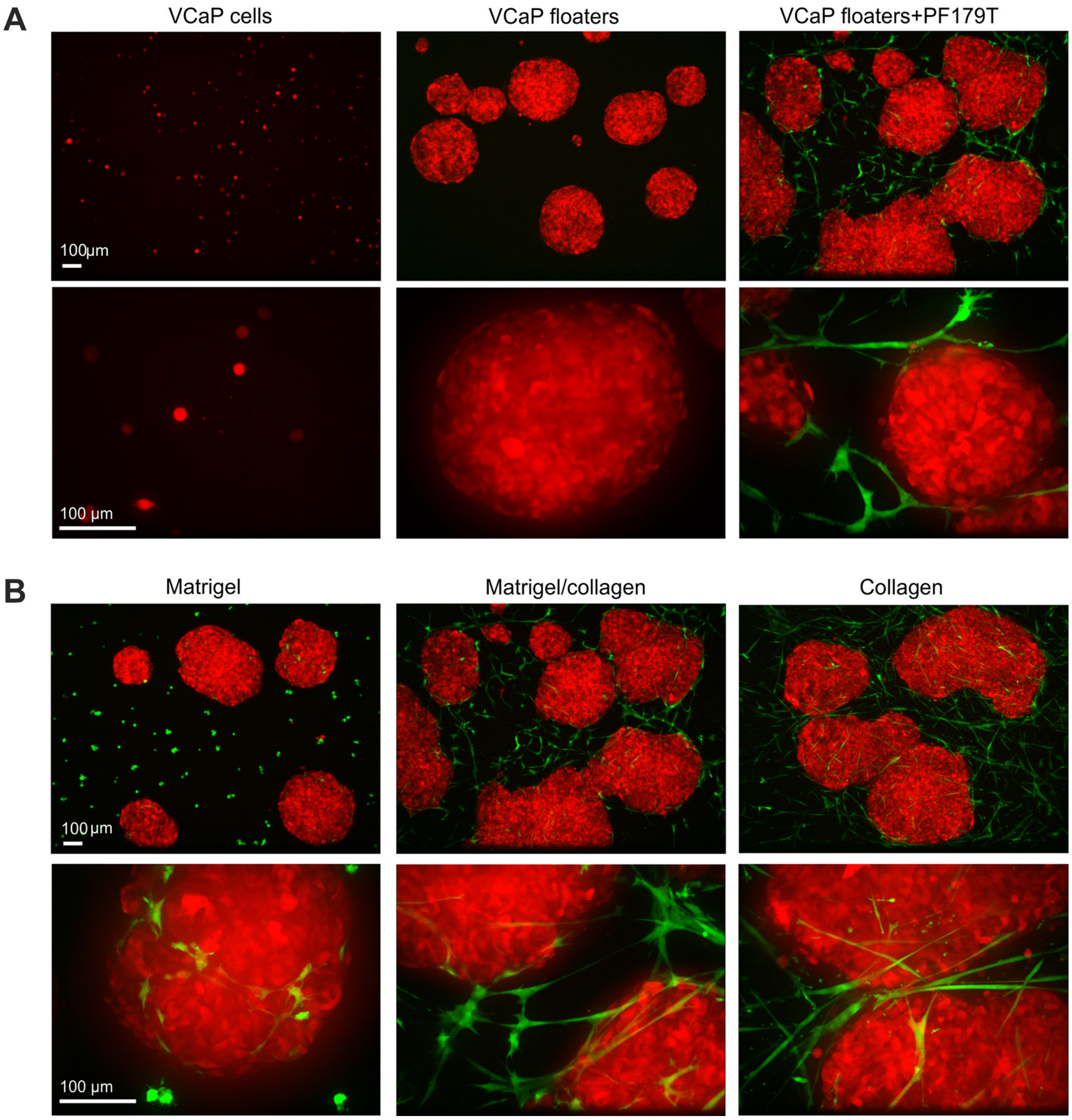

Supplementary Figure S7: Confocal imaging of RFP-VCaP cells and spheroids in 3D culture. A. Single VCaP cells do not proliferate in 3D culture, but remain alive (day 14), whereas pre-formed spheroids as mono- or in co-culture with CAFs grow rapidly. **B.** VCaP pre-formed spheroids were co-cultured with PF179T cells for 8 days in Matrigel, collagen and mixture. Red: dsRed-LNCaP, green: GFP-PF179T. Scale bar: 100 µm.

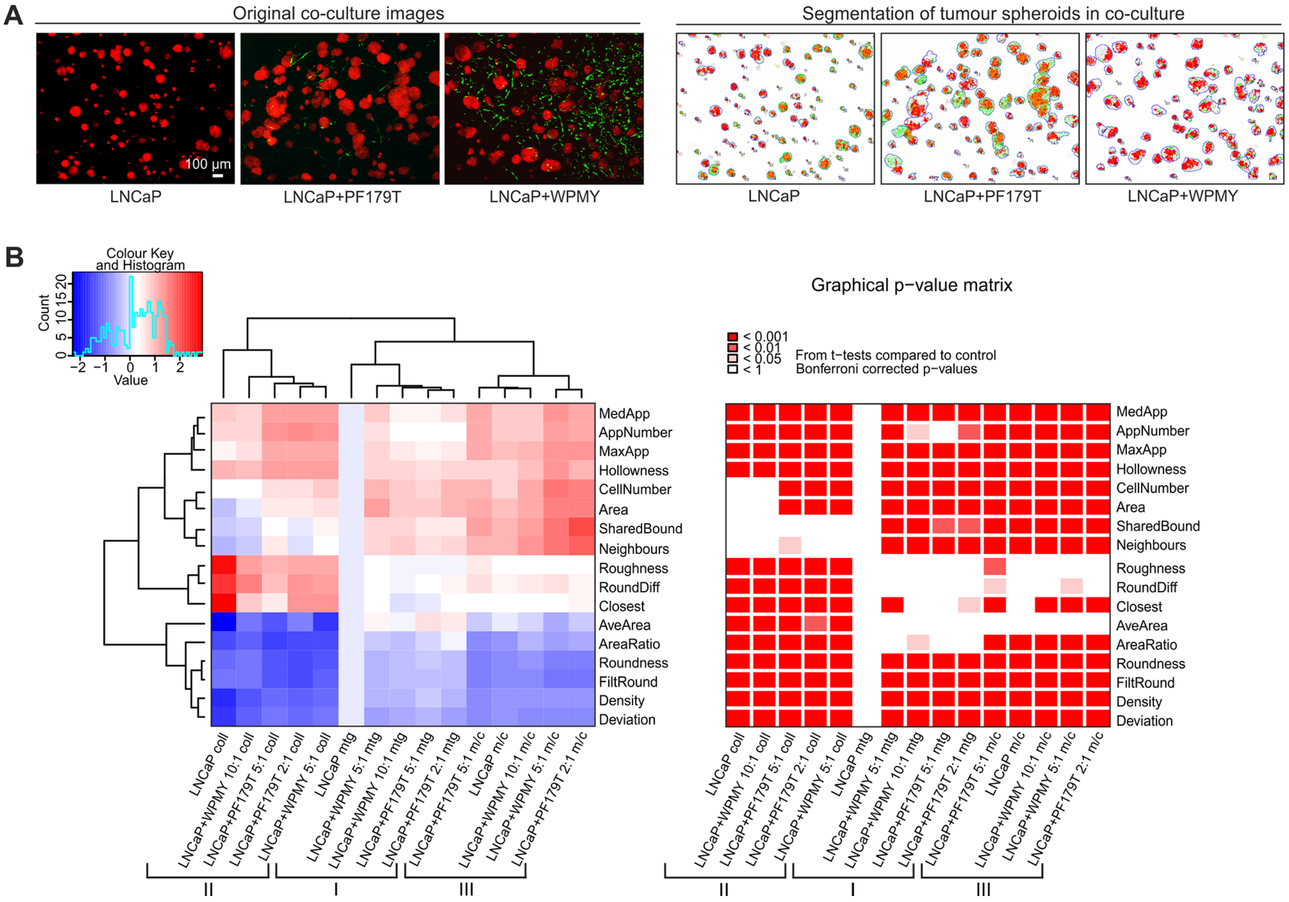

Supplementary Figure S8: Morphometric analysis of LNCaP organoids co-cultured with fibroblasts. A. Example confocal images of LNCaP organoids in mono- or co-culture (left panel), segmented by the automated image data analysis AMIDA software (right panel). Scale bar: 100 µm. **B.** Quantification of morphological changes in tumor cells in different cell ratios and ECMs. Three distinct groups of tumor phenotypes are marked with roman numerals (I = Matrigel, II = collagen, III = Matrigel/collagen mixture). Heatmap and graphical *P*-value matrix summarize the altered parameters from the AMIDA analysis. Values are color-coded as red (increased) and blue (decreased), relative to control. For explanation of morphometric parameters, see Supplementary Table S1. *P*-values are Bonferroni-corrected from *t*-tests and compared to DMSO control treatment (DMSO ctrl).

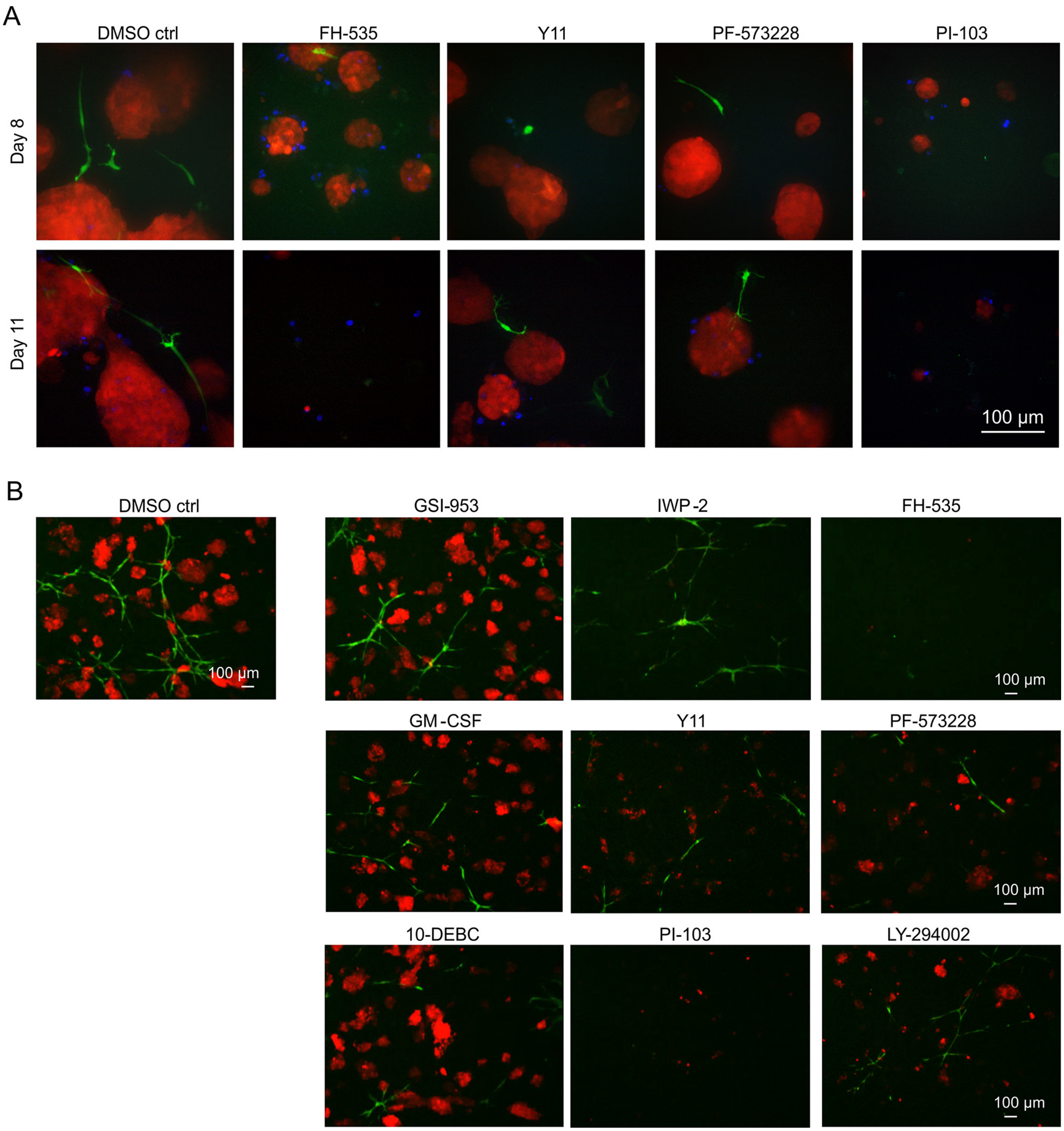

Supplementary Figure S9: Cytotoxicity testing and imaging of microtissues treated with small molecule inhibitors. A. Cytotoxicity was assessed using Draq7 (blue), which stains dead and permeabilized cells, at day 8 and day 11 of 3D co-culture. **B.** Representative end-point maximum projection spinning disc confocal images (5×) of 3D co-cultures cultured for 14 days in Matrigel/collagen mixture, treated with inhibitors (3 µM) or GM-CSF (0.3 ng/ml). Red: dsRed-LNCaP, green: GFP-PF179T (left panel). Scale bars: 100 µm.

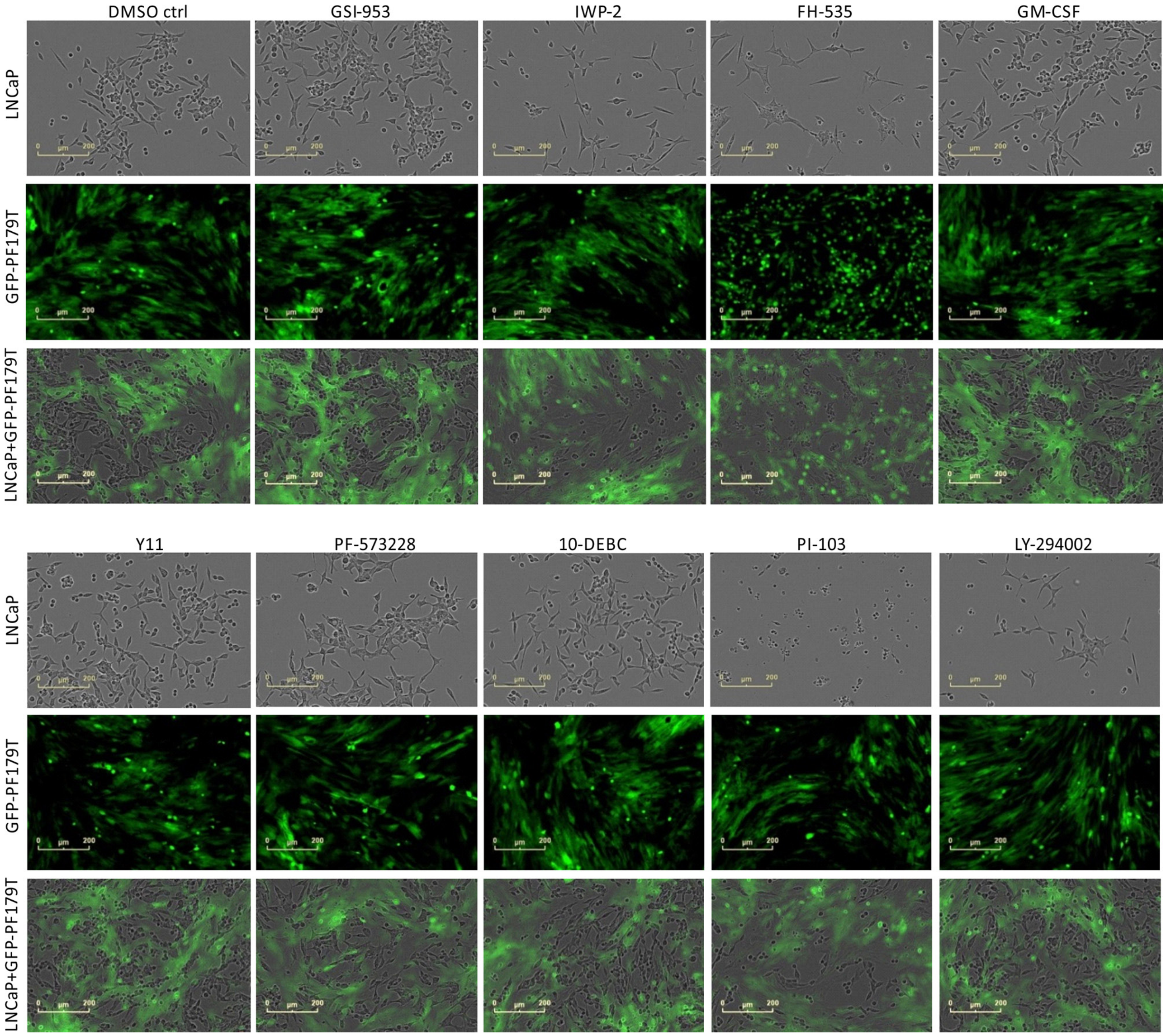

Supplementary Figure S10: Inhibition of WNT-, FAK- and PI3K-signaling in 2D mono- and co-cultures. Incucyte FLR live-cell imaging of LNCaP and PF179T cells alone or co-cultured in 2D, treated with inhibitors (3 μM) or GM-CSF (0.3 ng/ml), for 4 days. Upper panel: LNCaP cells alone, middle panel: PF179T cells alone, lower panel: co-culture of LNCaP and PF179T cells. Scale bar: 200 μm.

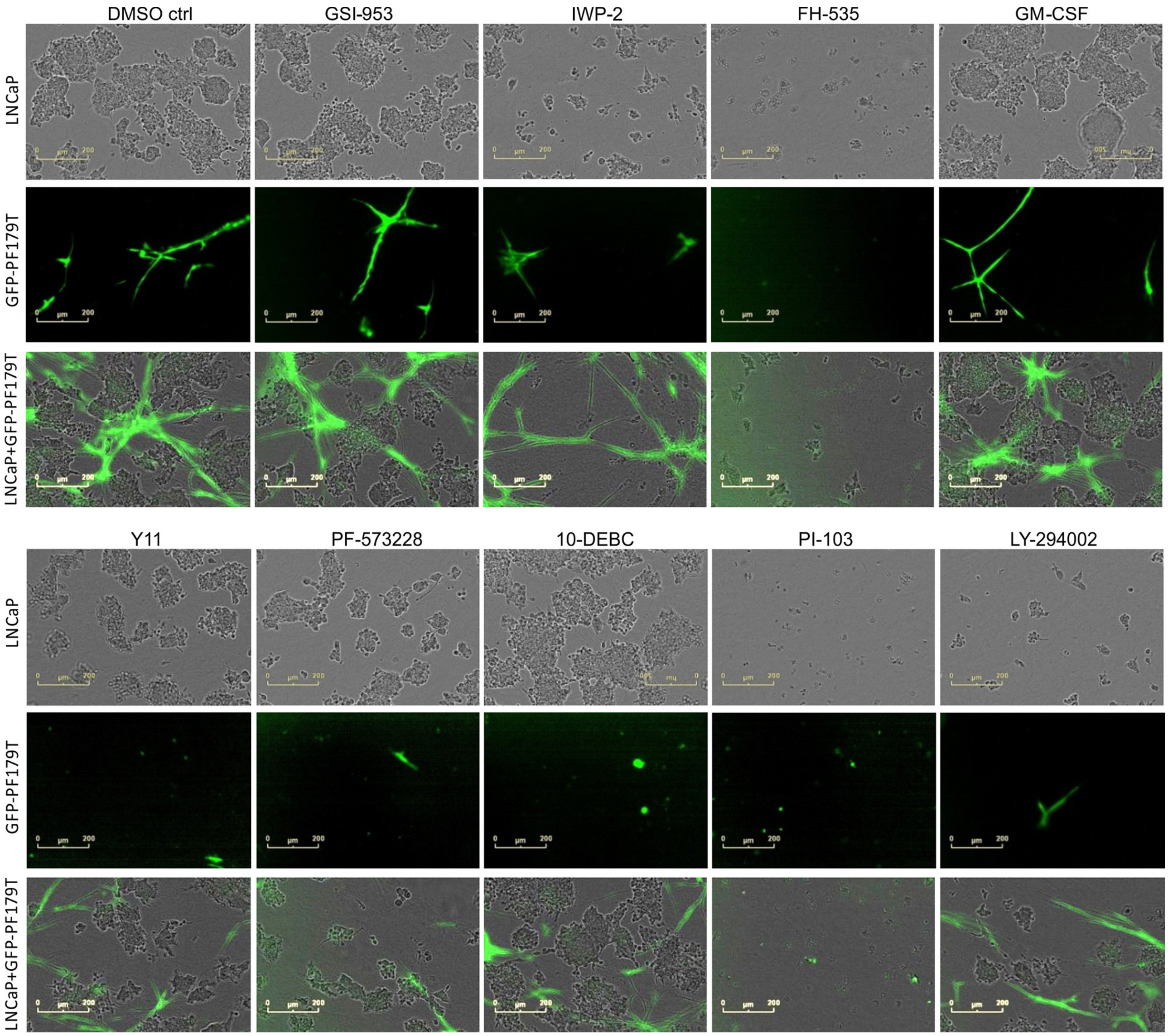

Supplementary Figure S11: Inhibition of WNT-, FAK- and PI3K-signaling in 3D mono- and co-cultures. Incucyte FLR live-cell imaging of LNCaP and PF179T cells alone or co-cultured in 3D, treated with inhibitors (3 µM) or GM-CSF (0.3 ng/ml), for 14 days. Upper panel: LNCaP cells alone, middle panel: PF179T cells alone, lower panel: co-culture of LNCaP and PF179T cells. Scale bar 200: µm.

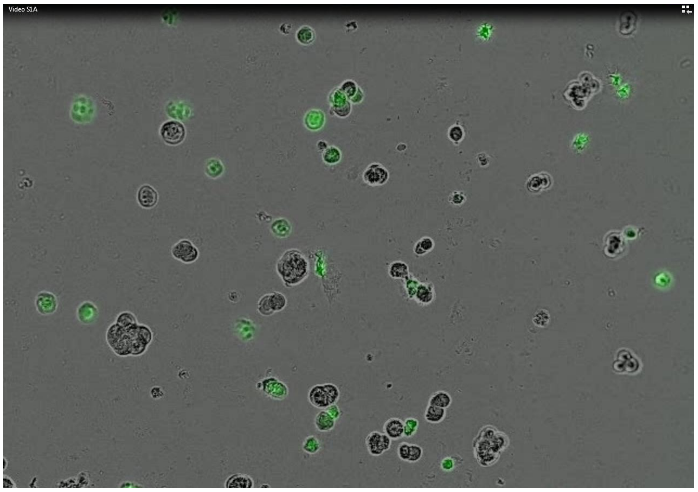

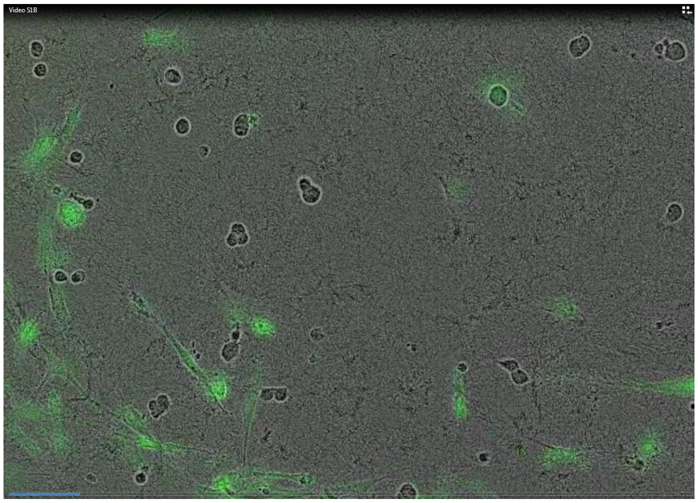

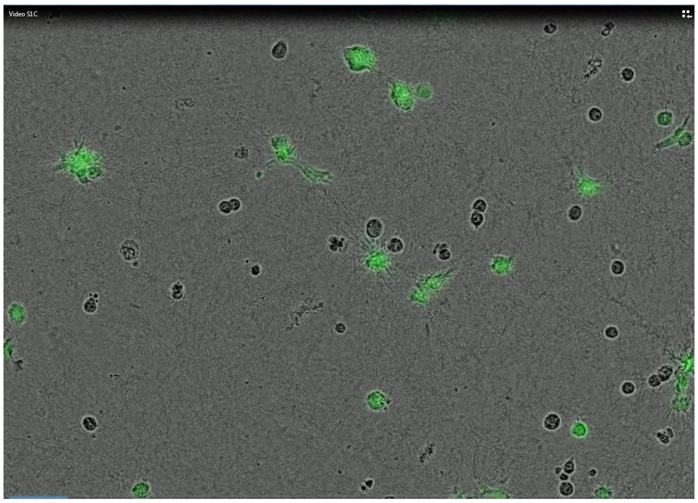

Supplementary Video S1: Collagen fibers are essential for fibroblast dynamics. Incucyte FLR real-time live image sequences of LNCaP (phase contrast) and GFP-PF179T CAF (green fluorescence) co-cultured for 14 days in **A.** Matrigel, **B.** collagen, and **C.** Matrigel/ collagen mixture.

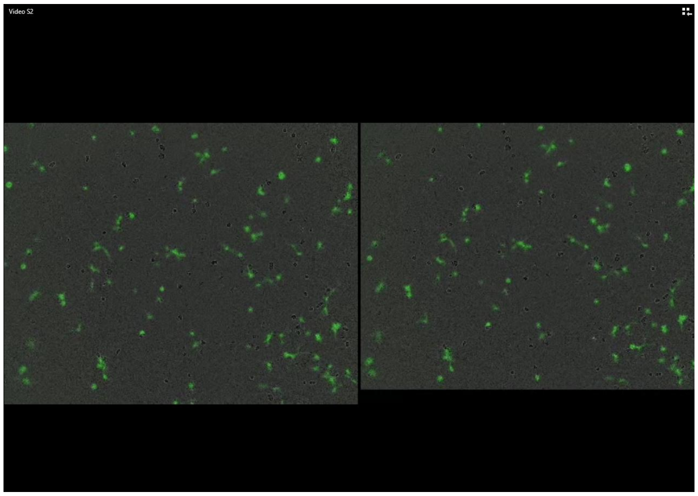

Supplementary Video S2: Image sequence stabilization. Filtered Incucyte FLR time-lapse sequence, stabilized using frame-by frame alignment, for robust analysis of CAF dynamics (phase contrast: LNCaP, green: PF179T).

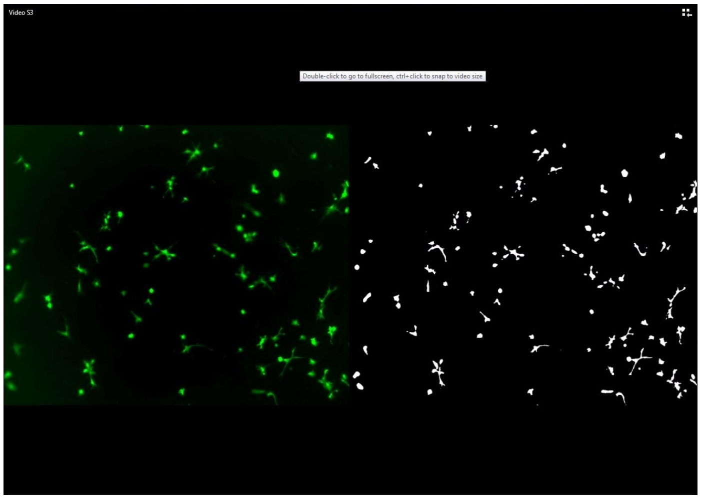

Supplementary Video S3: Segmentation of CAF cohorts in 3D co-cultures. Stabilized Incucyte FLR time-lapse sequence, segmented by local adaptive thresholding, for robust analysis of CAF dynamics (phase contrast: LNCaP, green: PF179T).

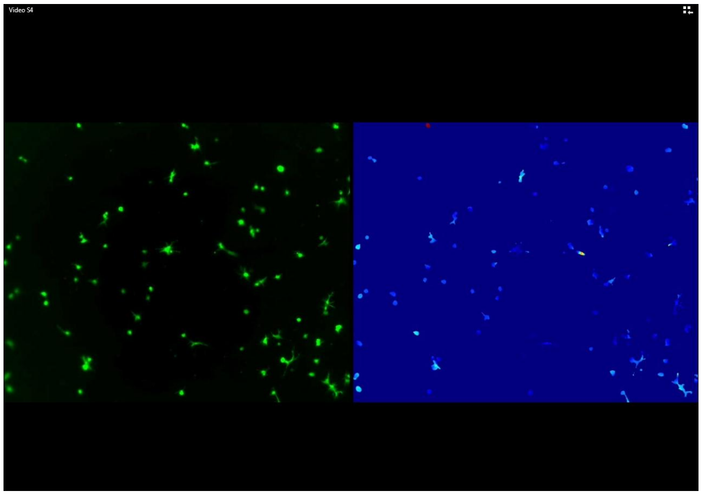

Supplementary Video S4: Motion analysis of CAF cohorts in 3D co-culture. Segmented Incucyte FLR time-lapse sequence used for CAF cohort motion analysis, implemented by dense optical flow estimation and application of the Farnebäck algorithm. Relative colors are encoded by colors (red: fast, blue: slow).

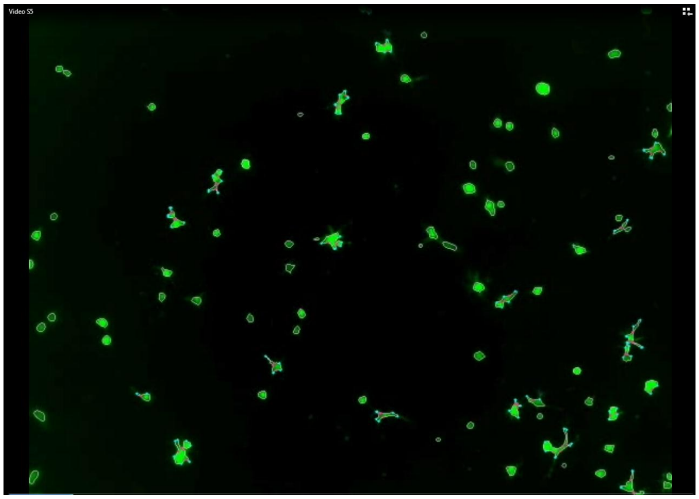

Supplementary Video S5: Morphological analysis of CAF cohorts in 3D co-culture. Segmented Incucyte FLR time-lapse sequence used for quantitative shape analysis, *i.e.* branching of CAF cohorts, examined by extracting the closed contour, and detecting the convex contour hull and convexity defects.

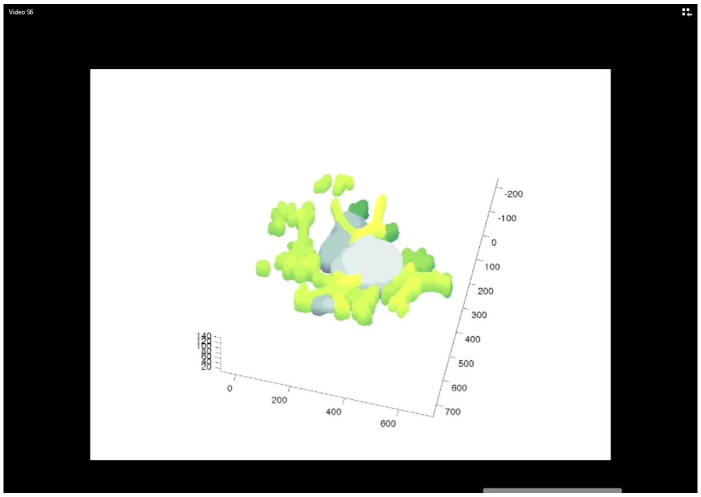

Supplementary Video S6: CAF cohorts are in contact with tumor organoids. Segmentation of LNCaP organoids co-cultured with PF179T CAFs in a 3D confocal image stack, illustrating that CAFs mainly surround but also make contact with the LNCaP organoids.

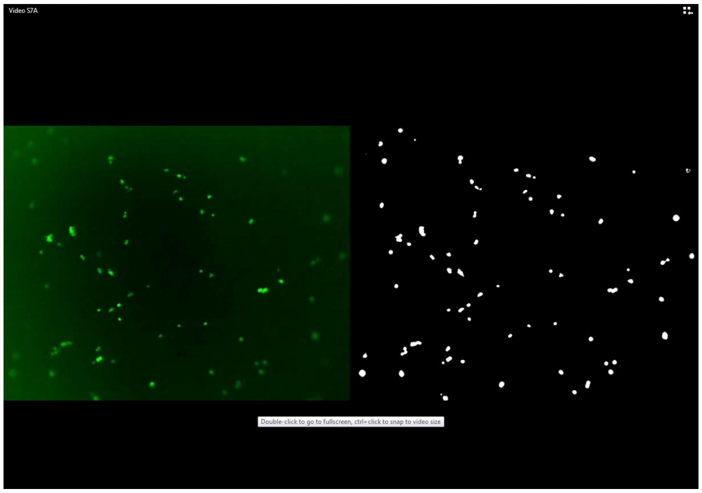

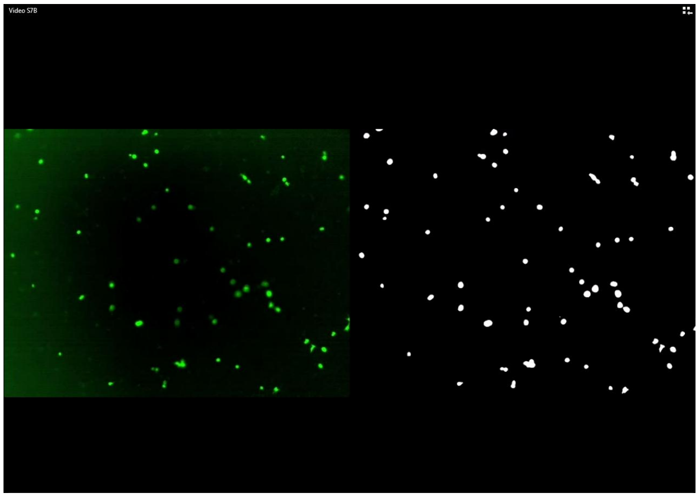

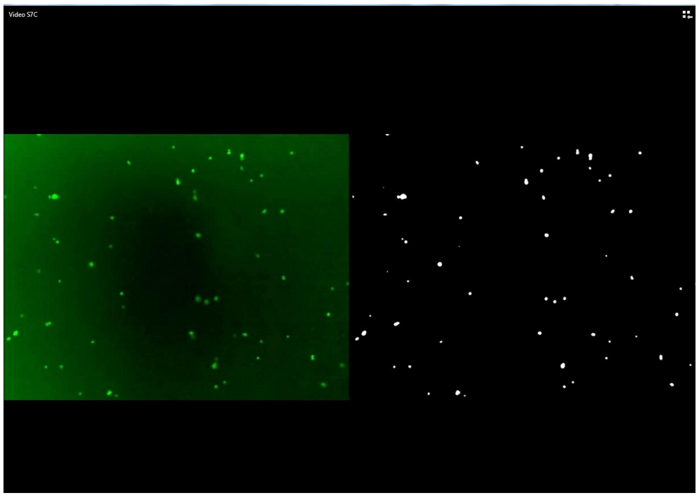

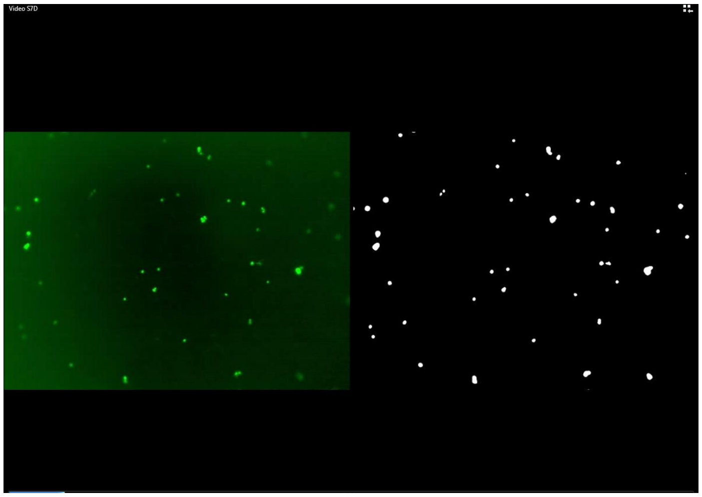

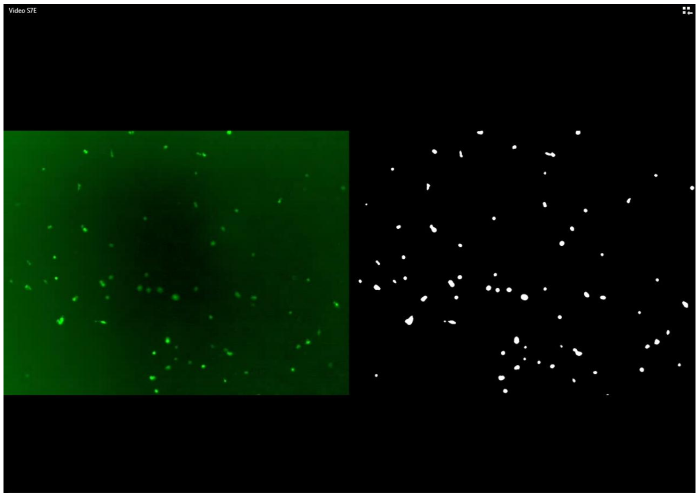

Supplementary Video S7: Small molecule inhibitors affect fibroblast dynamics. Live-cell imaging and segmentation of GFP-PF179T cells (green) co-cultured with LNCaP speroids (not visible). Cultures were treated with inhibitors at a concentration of 3 µM: **A.** DMSO control (ctrl), **B.** FH-535, **C.** PI-103, **D.** Y11, **E.** PF-373228, indicating that blocking of WNT/β-catenin, PI3K/mTOR and FAK signaling pathways significantly reduce CAF dynamics in 3D.

Supplementary Table S1: Morphometric parameters in the AMIDA image data analysis software

	Parameter	Explanation	Unit
**General**	Area	Area of the segmented structure	pixels
	Neighbors	The number of Neighboring structures touching the segmented structure	pieces
	SharedBound	The length of the shared boundary of all Neighbors of the structure	pixels
	Closest	The distance of the closest Neighbor of the segmented structure from the center point to the center point	pixels
	CellRatio	Cells to background ratio	ratio
**Morphological**	Roundness	Roundness of the segmented structure	%
	FiltRound	Filtered roundness of the segmented structure	%
	RoundDiff	Difference of the Roundness and Filtered Roundness	pp
	MaxApp	Estimate for the maximum length of appendages of the segmented structure	pixels
	MedApp	Estimate for the median length of appendages of the segmented structure	pixels
	Roughness	Roughness of the surface of the segmented structure	%
	AppNumber	Estimate for the number of appendages in the segmented structure	pieces
**Functional**	Density	Density of the segmented structure for each channel	gray levels/pix
	Deviation	Standard deviation of the segmented structure for each channel	no unit
	AreaRatio	Ratio of substructures of a certain color inside the segmented structure	%
	Hollowness	Estimate of the hollowness of the segmented structure for each channel	%
	CellNumber	Estimate of the number of cells in the red channel inside the segmented structure	pieces
	AveArea	Average area of the cells inside the segmented structure for each channel	pixels
